# Organic–inorganic supramolecular solid catalyst boosts organic reactions in water

**DOI:** 10.1038/ncomms10835

**Published:** 2016-02-25

**Authors:** Pilar García-García, José María Moreno, Urbano Díaz, Marta Bruix, Avelino Corma

**Affiliations:** 1 Instituto de Tecnología Química, UPV-CSIC, Universidad Politécnica de Valencia, Avenida de los Naranjos s/n, E-46022 Valencia, Spain; 2 Instituto de Química Física Rocasolano, CSIC, Serrano 119, 28006 Madrid, Spain; 3 King Fahd University of Petroleum and Minerals, PO Box 989, 31261 Dhahran, Saudi Arabia

## Abstract

Coordination polymers and metal-organic frameworks are appealing as synthetic hosts for mediating chemical reactions. Here we report the preparation of a mesoscopic metal-organic structure based on single-layer assembly of aluminium chains and organic alkylaryl spacers. The material markedly accelerates condensation reactions in water in the absence of acid or base catalyst, as well as organocatalytic Michael-type reactions that also show superior enantioselectivity when comparing with the host-free transformation. The mesoscopic phase of the solid allows for easy diffusion of products and the catalytic solid is recycled and reused. Saturation transfer difference and two-dimensional ^1^H nuclear Overhauser effect NOESY NMR spectroscopy show that non-covalent interactions are operative in these host–guest systems that account for substrate activation. The mesoscopic character of the host, its hydrophobicity and chemical stability in water, launch this material as a highly attractive supramolecular catalyst to facilitate (asymmetric) transformations under more environmentally friendly conditions.

## Abstract (web summary)

Coordination polymers and metal-organic frameworks are appealing synthetic hosts for mediating reactions. Here, the authors report a mesoscopic metal-organic structure that is shown to accelerate condensation reactions in water in the absence of acid or base, owing to the hydrophobic environment of the host.

## Graphical abstract

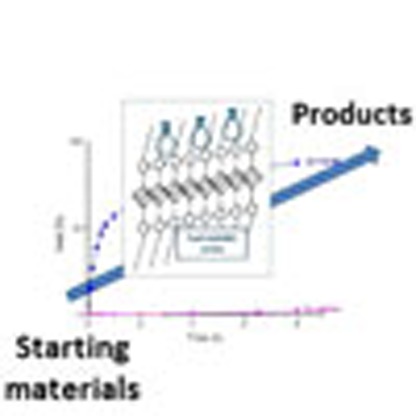

Metal-organic frameworks (MOFs) emerged as a new type of porous crystalline solids with tailorable structures and functions. The high versatility and possibilities that these materials offer are making them very attractive for specific applications such as gas storage^1^,^2^,^3^, separations^4^,^5^, luminescent^6^, conducting^7^, and magnetic materials^8^, drug delivering systems^9^ and catalysis^10^.

MOF-type metal-organic structures are frequently obtained as 3D frameworks based on the coordination binding between metallic nodes and rigid bi- or multi-podal linkers^11^,^12^. Preparation of modifiable layered metal-organic materials formed by individual sheets, spatially organized through electrostatic interactions is, however, not described in the literature, as far as we know. Only incipient studies related with the formation of MOF nanosheets by modulation of growth kinetics have been reported^13^. Nevertheless, the existence of 3D conventional MOFs which exhibited lamellar structural tendency in the spatial distribution of their structural building units could be indicative of the possibility to obtain metal-organic structures by assembly of lamellar units. In particular, from a topological point of view, this type of MOFs are based on infinite inorganic M-O-M chains, formed by only discrete metal clusters, separated by aryl dicarboxylate linkers perpendicularly located to individual metallic nodes alignment^14^,^15^. This topology can be observed in different well-known MOFs such as MIL-53(Al) (ref. 16), DUT-4, DUT-5 (ref. 17), DUT-8 (refs 18, 19), MOF-Zn-DABCO (ref. 20), Cu-(tpa)^21^ or MIL-68(Al) (ref. 22), in which it is possible to distinguish the 2D lamellar inorganic sub-networks.

Notably, many enzymes can promote chemical reactions via hydrophobic cavities wherein the cumulative influence of not only the active site in the enzyme but also the non-covalent bonds interactions account for substrate activation. Several synthetic host–guest systems have been used that copy these remarkable properties such as cyclodextrins^23^,^24^,^25^, cucurbiturils^26^,^27^, (metalla)crown ethers^28^, calixarenes^29^, carcerands^30^ and zeolites^31^. Solvation effects^32^,^33^ have also been studied and proved to account for rate acceleration in chemical reactions in such a way that approach enzyme-like catalysis. Lately, self-assembled hosts^34^,^35^,^36^,^37^ have demonstrated to function as ‘molecular flasks' to entail unusual reactions, or unique chemical phenomena. However, the development and engineering of larger and more sophisticated host structures that can incorporate several reaction components is still a challenge. An appealing strategy for supramolecular catalysis involves the design of a host system able to accommodate a given (ideally any) homogeneous catalyst and also the two reacting components, facilitating the reaction of the two partners even further and/or allowing for more environmentally friendly reaction conditions, such as lower reaction temperature, lower homogeneous catalyst loading or the use of water as a green solvent. Here we report the initial steps towards this design plan. Taking into consideration the lamellar tendency of MIL-53 structures, we prepare a novel metal-organic-type material, Al-ITQ-HB, which is based on ordered individual aluminium clusters-type sheets, similar to the sub-networks detectable in MIL-53-type materials. Key in our design is the use of a specific organic spacer, 4-heptylbenzoic acid (HB), with only one reactive carboxylate group that interacts with inorganic metallic nodes through stable coordination bonds. This particular organic spacer contains hydrocarbonated tails, which control the separation between metallic nanosheets, inhibit the 3D growth observed in conventional MOFs, favouring the formation of mesoscopic non-ordered phases with well-defined metal-organic monolayers^38^. We use this material as a supramolecular hybrid structure in cases where the hydrophobic part acts to concentrate reactants and acts as hydrophobic pockets wherein molecules are activated when performing reactions even in aqueous media. The material markedly accelerates condensation reactions in water in the absence of acid or base catalyst, as well as organocatalytic Michael-type reactions that also show superior enantioselectivity when comparing with the host-free transformation.

## Results

### Synthesis and characterization of the metal-organic material

**Figure 1:**
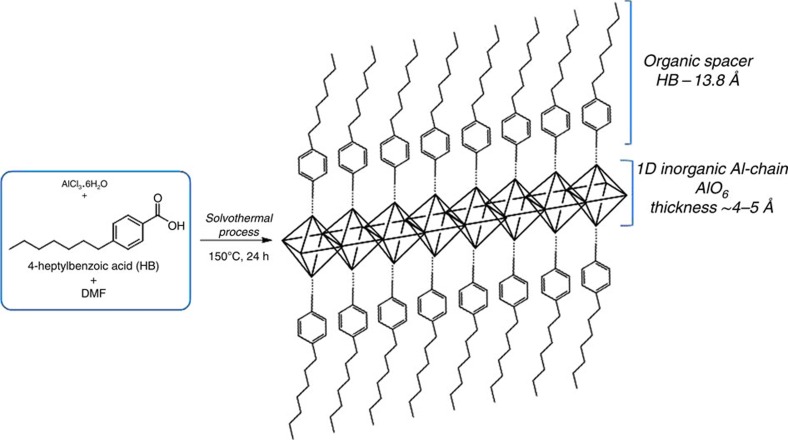
Synthetic route followed to obtain mesoscopic-type hybrid material. The synthetic procedure was replicated >10 times. Representation of Al-ITQ-HB as individual organic–inorganic nanosheet is shown. The individual nanosheets would be formed by consecutive corner-sharing octahedral (AlO_6_) units, conforming 1D inorganic chains, which are separated by alkyl benzene monocarboxylate ligands, located on both sides of metallic nodes. So, the coordinative association between inorganic chains and organic spacers in the opposite sides of metallic nodes forms each individual organic–inorganic layer which would be the basis of this type of metal-organic mesoscopic structures. DMF, dimethylformamide.

We started with a MIL-53 (Al)-type material and decided to synthesize 2D nanosheets (see Fig. 1). This was achieved by inhibiting the 3D growth in the MIL-53 (Al) crystals and favouring the formation of mesoscopic phases that fitted our catalytic purposes. In our case, *para*-heptyl benzene monocarboxylate compound (HB) was used as molecular spacers (Fig. 1). This organic spacer was utilized instead of conventional rigid aryl dicarboxylate linkers, such as terephthalic acid, normally used in the preparation of the major part of MOF-type structures. Solvothermal process in the presence of aluminium chloride, dimethylformamide and HB as inorganic source, solvent and organic spacers, respectively, facilitated the preparation of the mesoscopic hybrid material whose walls were based on metal-organic layers with octahedral aluminium units separated by the hydrocarbonated tails acting as spacers, perpendicularly located to inorganic nodes. Representation of individual organic–inorganic layers is also drawn in Fig. 1, highlighting the role of organic spacers (HB) as effective growing inhibitors of the standard 3D metal-organic structure.

The X-ray diffraction pattern of the as-synthesized hybrid material Al-ITQ-HB (Supplementary Fig. 1), showed that mesoscopic organization could be achieved being clearly observable a (*100*) low-angle diffraction peak (*d*_*100*_=∼35 Å) which is characteristic of mesoporous solids with short-range hexagonal ordering. However, *(100)* diffraction band could also be distinctive of 2D lamellar frameworks formed by individual sheets perpendicularly disposed to axis-*a*, indicating that a certain regularity would exist in the separation (basal space of ∼35 Å) between successive piled organic–inorganic nanosheets. In any case, the analysed hybrid solid did not exhibit a very high structural order, as deduced from the low intensity and broadness of the low-angle *(100)* diffraction band together with the absence of repetitive *(h00)* diffraction bands.

**Figure 2:**
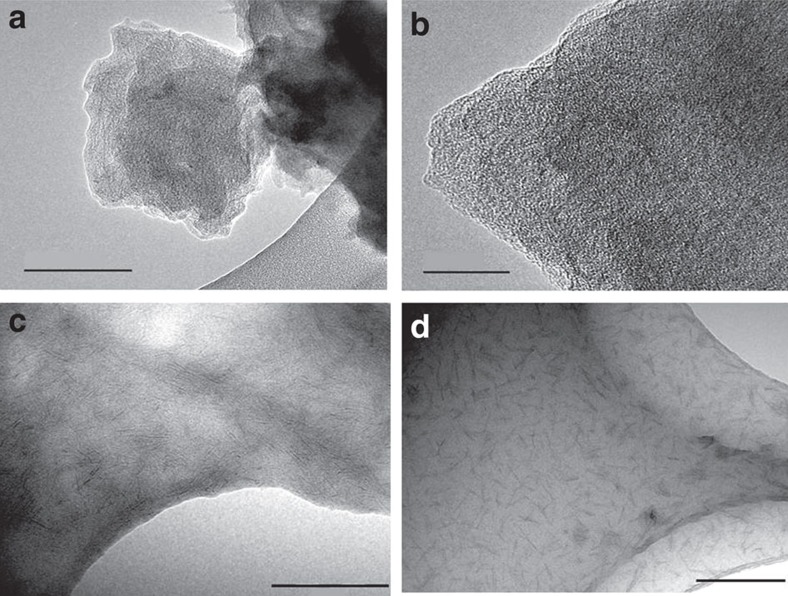
HRTEM images of Al-ITQ-HB sample. (**a**) As-synthesized material. Scale bar, 100 nm. (**b**) As-synthesized material. Scale bar, 20 nm. Mesoporous cavities are clearly detected. A pore-size distribution around 20 Å in diameter is observed. (**c**,**d**) material after post-synthesis treatment with dichloromethane generating a stable solution. Scale bars, 200 nm. The high hydrophobicity exhibited by Al-ITQ-HB solid, due to the elevated organic content, facilitated the interlayer penetration of solvent molecules with the consequent delamination effect, being observable individual nanolayers dispersed in the stable solution.

The mesoscopic long-order organization was observed from TEM microscopy, being clearly detected the presence of mesoporous which were distributed along plate-like crystals (Fig. 2a,b, Supplementary Fig. 2). The micrographs also showed the existence of areas formed by short sheets which would evidence the dual nature of the Al-ITQ-HB materials, that is, lamellar and mesoporous due to the presence of metal-organic nanolayers, probably assembled around non-ordered mesocavities. However, the low structural periodicity exhibited by this type of hybrid solids along the channel direction would hinder the easy detection of the lamellar sub-domains from electronic microscopy. Despite that, the lamellar nature of the hybrid sample was confirmed through the exfoliation phenomenon achieved in presence of non-polar solvents (Fig. 2c,d, Supplementary Fig. 3).

From the elemental CHNS analysis (Supplementary Table 1), it was possible to estimate the amount of organic content present in the hybrid material, Al-ITQ-HB. The results indicated that the organic counterpart contribution was around 40 wt%, corresponding to alkylaryl fragments. Furthermore, the practical absence of nitrogen content in the solid showed that most of the dimethylformamide, used as solvent during the solvothermal synthesis, was removed during the successive washing steps.

The weight loss with temperature and the corresponding derivative (TGA and DTA curves; Supplementary Fig. 4) for the mesoscopic metal-organic hybrid material allowed establishing, not only the amount of organic spacers incorporated in the Al-ITQ-HB solid, but also their hydrothermal stability. After elimination of the major part of hydration water and residual dimethylformamide (both detected at around 80–150 °C), it was possible to see a main weight loss, between 450 and 600 °C, assigned to the presence of *para*-alkyl benzene monocarboxylate molecules (HB) used as spacers. It is important to remark the weight loss observed between 250 and 400 °C assigned to the decomposition (dehydration phenomenon) of AlO_4_(OH)_2_ units present in the 1D inorganic chains^39^, which would confirm again the presence of lamellar organic–inorganic sub-domains integrated into the mesoscopic framework. In this temperature range, contribution of alkyl chains from incorporated organic linkers HB, would also be included into this weight loss.

The ^13^C CP/MAS NMR spectrum of Al-ITQ-HB (Supplementary Fig. 5B) confirms the total integrity of the organic spacer (HB) after the solvothermal synthesis processes since all carbon atoms were unequivocally assigned. In addition, the presence and integrity of alkyl benzene monocarboxylate units located in the walls of Al-ITQ-HB solid was confirmed by infrared spectroscopy (Supplementary Fig. 6). On the other hand, ^27^Al MAS NMR spectrum (Supplementary Fig. 7) of the Al-ITQ-HB sample, presented a defined peak at 6.7 p.p.m. chemical shift that is characteristic of aluminium (oxo)hydroxide species, showing a regular octahedral environment of metallic atoms. This result would confirm the formation of 1D inorganic chains of (AlO_6_) octahedral units in the assembled organic–inorganic nanolayers^40^.

Textural properties for Al-ITQ-HB solid were analysed from Argon adsorption isotherm (Supplementary Fig. 8), being estimated a reduced surface area and porous volume (S_BET_ ∼35 m^2^ g^−1^, V_TOTAL_ ∼0.11 cm^3^ g^−1^) due to, probably, the big amount of organic content (∼40 wt%) present in the hybrid material that hinders the correct gas adsorption along the mesocavities. The marked hydrophobic character of the material would reinforce this phenomenon. However, the Hörvath–Kawazoe pore-size distribution (Supplementary Fig. 9) showed that the majority of pores were centred at ∼20–25 Å, this value being coincident with that estimated from electronic microscopy, remarking the mesoscopic nature of the Al-ITQ-HB material.

CO adsorption at low temperature (100 K) was monitored by FTIR spectroscopy to gather information relating Lewis acidic sites in both samples, MIL-53 (Al) and Al-ITQ-HB (Supplementary Figs 10 and 11). An absorption band is observed at 2,158 cm^−1^ that already indicates that the Lewis acidity of these materials is very weak. This being confirmed by the fact that a complete disappearance of this band is observed at 100 K by subsequent treatment of the sample in vacuum.

### Knoevenagel condensation reaction

The extremely flexible hybrid material obtained could act as a supramolecular catalyst in cases where the hydrocarbonated alkylaryl spacers would constitute hydrophobic pockets when working in aqueous media. Indeed, we found that Al-ITQ-HB efficiently promotes the Knoevenagel condensation of 2-naphthaldehyde (**1a**) with Meldrum's acid (**2**) in H_2_O:CHCl_3_ in the absence of base or acid catalysts. Under the optimized conditions (Supplementary Tables 2 and 3), the condensation product (**3a**) was formed in 91% yield after 10 h (Table 1, entry 1). The reaction conditions are very mild (neutral, room temperature) and the reaction proceeds catalytically (30 mol% of Al-ITQ-HB) as the product easily diffuse through the mesoporous channels liberating the host to embrace another pair of reacting components. Without Al-ITQ-HB, **1a** gave only small amount of the **3a** (4%) under otherwise identical conditions. Carrying out the reaction at higher molarity of CHCl_3_ as the sole solvent in the absence of Al-ITQ-HB, afforded the product in low yield of 10%, (Supplementary Table 4), allowing us to conclude that rate enhancement is not a mere consequence of the concentration rise. Al-ITQ-HB is not simply increasing molarity of reagents but also promotes the condensation in water. Control experiment with conventional MIL-53(Al) showed barely formation of product (17%). The hydrophobic cavity of Al-ITQ-HB is, therefore, crucial for the reaction because the individual MOF components Al(OH)(C_2_H_3_O_2_)_2_ or HB acid did not promote the transformation substantially (Supplementary Table 4). Micellar catalysis was also tested by using the equivalent amount of sodium 4-heptylbenzoate that resulted in low product formation (19% yield under otherwise identical conditions, Supplementary Table 4). Rate acceleration appeared more obvious when following the Knoevenagel condensation reaction over time (Supplementary Figs 12 and 13). We observe that initial reaction rate in the case of Al-ITQ-HB in the presence of water (Supplementary Fig. 12) is 540 times higher than when no additive is used. Initial reaction rate is also superior for Al-ITQ-HB versus utilization of HB acid (54 times higher), sodium 4-heptylbenzoate (54 times higher) or MIL-53 (Al) (216 times higher). Rise in reaction rate for Al-ITQ-HB was also observed in organic solvents (such as CHCl_3_, Supplementary Fig. 13) wherein initial rate is 105 times superior when using Al-ITQ-HB versus no additive utilization.

Substrate scope of the Knoevenagel reaction is shown in Table 1 (see also Supplementary Methods). Under the very mild conditions, a variety of aromatic aldehydes underwent efficient condensation when using Al-ITQ-HB. Control experiments without Al-ITQ-HB showed that reaction hardly occurred. Once again, 3D MIL-53 (Al) showed no relevant conversion to the **3a** highlighting the prominent role of the hydrophobic pocket and demonstrating that substrate activation is not a mere consequence of Lewis acidic activation by the framework, or the influence of *π*-stacking interaction of the starting aromatic aldehyde with the framework. The method proceeded better with aromatic aldehydes having electron donating groups (Table 1, entry 4), although rate acceleration is observed in all cases.

After completion of the reaction, the supramolecular catalyst Al-ITQ-HB could be recovered by simple centrifugation and can be reused for the next run with a slight deterioration of the catalytic activity (72% conversion). PXRD showed that the recycled Al-ITQ-HB maintain its mesoscopic nature after use, being observable the characteristic low-angle diffraction band (Supplementary Fig. 14).

### Multicomponent reaction

**Figure 3:**
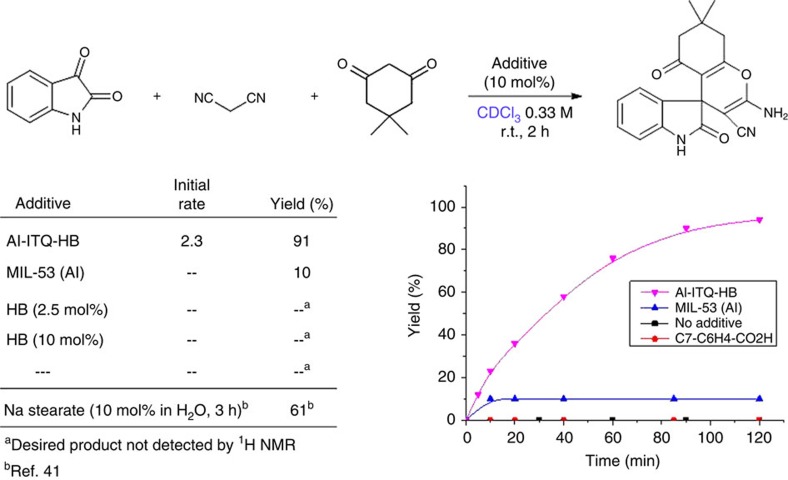
Al-ITQ-HB catalysed multicomponent reaction for the synthesis of spirooxindole product. Reaction conditions: isatin (0.2 mmol), dimedone (0.2 mmol), malononitrile (0.2 mmol) and additive (10 mol% except noted) in CDCl_3_ (0.33 M) at room temperature. ^1^H NMR yields (d6-DMSO) are reported (internal standard: Ph_3_CH).

The activity of Al-ITQ-HB was also tested in the multicomponent reaction of isatin, dimedone and malononitrile for the synthesis of the spirooxindole product (Fig. 3). This transformation proceeds under micellar catalysis in water by means of sodium stearate (10 mol%) (ref. 41). We found out that Al-ITQ-HB efficiently promoted the three-component reaction in an organic solvent such as chloroform, while the product is barely formed when using conventional MIL-53 (Al) and not detected by ^1^H NMR after 2 h reaction time when using HB acid or no additive at all (Fig. 3).

While micellar catalysis by means of sodium stearate gave comparable results as our Al-ITQ-HB on the bases of initial reaction rates (Supplementary Fig. 16), the attained yield after 2 h reaction time is higher for Al-ITQ-HB. Once again, in the presence of water, Al-ITQ-HB probed rate enhancement as compared with the MOF-free reaction under otherwise similar conditions (Supplementary Fig. 17). Superiority of Al-ITQ-HB is also shown versus utilization of the 3D MIL-53 (Al) or HB acid (Supplementary Fig. 17).

### Organocatalytic Michael-type reactions

**Figure 4:**
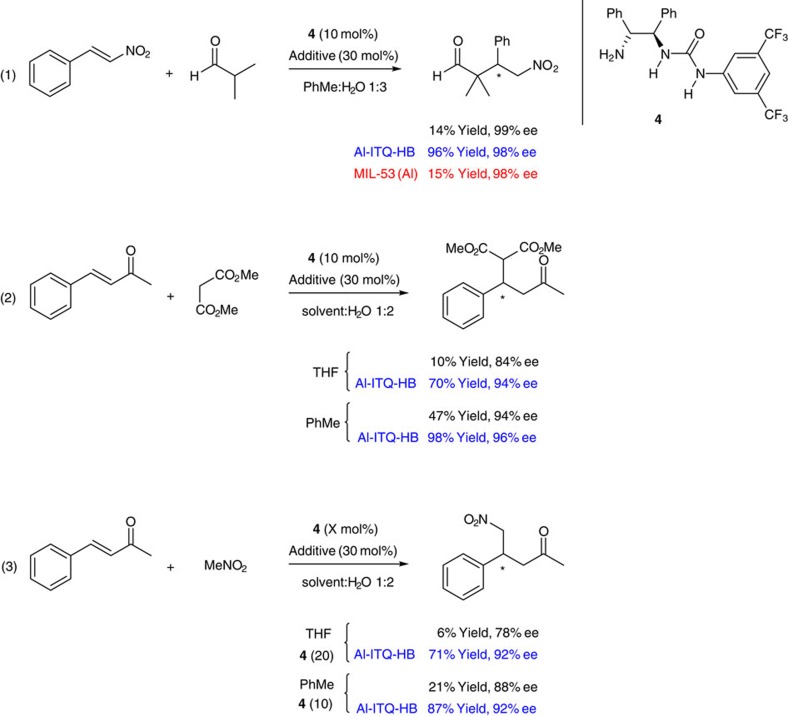
Enantioselective organocatalytic Michael reactions promoted by Al-ITQ-HB. Organocatalyst **4** was used in the amount of 10 mol% in all cases except noted. Additives used were Al-ITQ-HB (results highlighted in blue) or MIL-53 (Al) (results highlighted in red) in the amount of 30 mol%. All the experiments were performed at room temperature for 24 h. Yields are the average of at least two runs.

Given the excellent results attained in rate acceleration in the above condensation reactions and the mesoscopic nature of Al-ITQ-HB, we envisioned that this framework could also be applied to facilitate asymmetric organocatalytic reactions. Organocatalysis have become a very productive field or research that well complements metal catalysis and biocatalysis in offering new opportunities for (enantio)selective transformations^42^. Probably, the main limitations in organocatalysis are the fact of low turnover numbers and slow reaction rates often affecting procedures. As an example, Michael addition of isobutyraldehyde to nitrostyrene catalysed by several primary amine bifunctional organocatalysts (similar to **4** in Fig. 4) have shown to require loadings of 20–30 mol%, to achieve good to high product formation after relatively long reaction time of 2 days (refs 43, 44). In particular, 10 mol% of bifunctional amino-urea catalyst **4** provided the addition product in 14% yield and high enantioselectivity after 24 h reaction time (see equation 1 in Fig. 4). Remarkably, the combination of organocatalyst **4** with our supramolecular host catalyst Al-ITQ-HB provided substantial rate acceleration while maintaining the same degree of asymmetric induction. By this means, high yield (of 96%) can be achieved with relatively low organocatalyst loading, at room temperature, without the aid of noxious additives and after reasonable reaction time. Further analysis of the Al-ITQ-HB mediated reaction proved the role of the hydrophobic pocket in the host catalyst. Similar trends are observed here as in the case of the above depicted Knoevenagel condensation reaction. The presence of additives such as 1-phenylheptane, HB acid or MIL-53(Al) had no influence on the reaction rate (Supplementary Table 5). While host catalyst, Al-ITQ-HB, also shows rate enhancement in bare organic solvents such as toluene and CH_2_Cl_2_, higher product yields are attained in the presence of water evidencing the interplay of the hydrophobic effect that accounts partly for the observed efficiency. It was found that micellar catalysis exerted similar effect as our hydrophobic Al-ITQ-HB (Supplementary Table 5). However, while micellar medium required the so called critical micellar concentration to be operative, with our system the rate enhancement is observed even at low host catalyst load (Supplementary Table 5). The use of brine as reaction media instead of plain water showed advantage in the host-free reaction, although it did not provide further reaction acceleration in the presence of Al-ITQ-HB.

Following the organocatalytic Michael-type reaction over time shows the magnitude of the rate acceleration (Supplementary Fig. 18). We observe an important increase in the reaction rate in the case of Al-ITQ-HB versus utilization of HB acid, sodium 4-heptylbenzoate or no catalyst at all.

The observed efficiency achieved by combining host Al-ITQ-HB with the chiral bifunctional organocatalyst prompted us to evaluate the generality of this effect in other organocatalytic asymmetric transformations. Indeed, analogous rate enhancement is observed in the Michael addition of dimethylmalonate to 4-phenyl-3-buten-2-one (see equation 2 in Fig. 4). What it is more, higher enantioselectivity is observed when performing the reaction in the presence of host Al-ITQ-HB, evidencing that the supramolecular catalyst is indeed hosting the reacting 3-component system with non-covalent bond interactions playing a decisive role in rate acceleration and asymmetric induction. Furthermore, supramolecular host catalyst Al-ITQ-HB showed rate as well as enantioselectivity enhancement in the Michael addition of nitromethane to 4-phenyl-3-buten-2-one (see equation 3 in Fig. 4), establishing a considerable generality for the hydrophobic host system to facilitate organic reactions in aqueous media.

### NMR spectroscopy study of host–guest interactions

Intrigued by the way of action of solid Al-ITQ-HB in exerting the observed rate acceleration in the varied reactions shown above, we completed this study with information from techniques that provide a direct and detailed view on the underlying interactions at the molecular level. Solution NMR spectroscopy could provide such a view since intermolecular interactions can be studied through their effect on the chemical shift, relaxation times and translational diffusion coefficients of the various species. In the case of protein receptors and ligand binding, specific NMR approaches are routinely applied to provide information about the binding process and conformations. Due to the high flexibility of our solid Al-ITQ-HB and its high organic content, we wondered whether such solution NMR-based approaches could also be utilized in our heterogeneous system for the *in situ* characterization of the molecular interactions. In fact, 2D NOESY NMR experiments were carried out on suspension mixtures of naphthaldehyde **1a**+Al-ITQ-HB, organocatalyst **4**+Al-ITQ-HB and nitrostyrene+Al-ITQ-HB (Supplementary Figs 19, 20 and 21, respectively). In all three cases, it is shown through space correlation between the aromatic protons of the substrates **1a**, **4** and nitrostyrene with the hydrocarbon chain of Al-ITQ-HB. What it is more, changes in the chemical shifts and an increase in signal linewidths are observed in the ^1^H NMR spectrum of organocatalyst **4** (in CDCl_3_) when forming the suspension mixture with Al-ITQ-HB (Supplementary Fig. 22) evidencing the interactions between both systems and probably since Al-ITQ-HB may be disrupting the self-aggregation of the organocatalyst.

**Figure 5:**
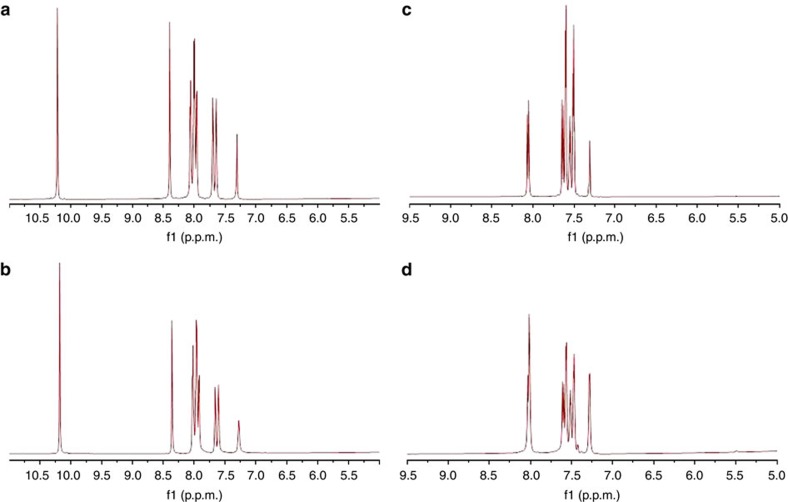
STD spectra (STD_off_—STD_on_) of substrate and Al-ITQ-HB suspensions. (**a**) ^1^H NMR reference spectrum of naphthaldehyde **1a** and Al-ITQ-HB suspension. (**b**) STD NMR of naphthaldehyde **1a** and Al-ITQ-HB suspension with saturation of methyl signal (0.9 p.p.m.) of Al-ITQ-HB. All of the naphthaldehyde resonances appear in the STD experiment with equal intensity as in the reference ^1^H NMR demonstrating that naphthaldehyde is indeed binding to Al-ITQ-HB. (**c**) ^1^H NMR reference spectrum of nitrostyrene and Al-ITQ-HB suspension. (**d**) STD NMR of nitrostyrene and Al-ITQ-HB suspension with saturation of methyl signal (0.9 p.p.m.) of Al-ITQ-HB. All of the nitrostyrene resonances appear in the STD experiment with equal intensity as in the reference ^1^H NMR demonstrating that nitrostyrene is indeed binding to Al-ITQ-HB.

Saturation transfer difference (STD) NMR spectroscopy is a powerful technique to investigate ligand binding to proteins^45^. Working with an excess of ligand, it consist in saturating some resonances of the protein target in the on-resonance experiment taking care not to affect the ligand resonances. This selective saturation subsequently spreads through the entire network of dipolar-coupled protons in the protein via spin diffusion. When a ligand binds to the protein, part of the saturation is transferred onto its protons. Since each protein undergoes multiple binding events during the saturation time, a sizable fraction of the ligands is affected, leading to a reduction of the ligand resonance intensity. This is easily characterized by subtracting a reference, the off-resonance experiment wherein saturation is applied outside the frequency range where ligand and protein resonances occur. The difference spectrum, therefore, yields non-zero intensities only for binding ligands. Furthermore, as ligand protons in close contact with the protein receive more saturation than more distant ones, the relative intensity of the ligand resonances can be interpreted in terms of a binding epitope. Despite the broad information that this technique could provide in supramolecular systems, it has been almost exclusively utilized in protein–ligand studies and only a few studies have been performed on synthetic hosts^46^,^47^. Here we demonstrate the potential of STD NMR for the investigation of interactions between hybrid metal-organic Al-ITQ-HB and several substrates. A suspension mixture of **1a** and Al-ITQ-HB was analysed (Fig. 5a). Saturation was performed at the resonance of the methyl group (0.9 p.p.m.) in the Al-ITQ-HB far away from resonances of aromatic protons of naphthaldehyde, whereas off-resonance frequency was −150 p.p.m. (Supplementary Methods). Pulse sequences followed were those reported in literature for protein–ligand systems^48^. Figure 5b shows the result of the STD experiment to the mixture shown in Fig. 5a. All of the naphthaldehyde resonances appear in the STD experiment with equal intensity as in the reference ^1^H NMR (Fig. 5a) demonstrating that naphthaldehyde is indeed binding to the supramolecular host catalyst. The major difference here with respect to the protein–ligand systems relays on the fact that we do not have a one-molecule site that binds to one protein-binding site, but rather a hydrophobic surface area that is large, with respect to the individual molecules, providing many locations and modes for binding. Similar results were attained when the binding substrate chosen was nitrostyrene (Fig. 5c=reference ^1^H NMR and Fig. 5d=STD NMR experiment). Further details on this study and blank experiments are shown in the Supplementary Figs 23–27. These results show that non-covalent interactions are operative in these host–guest systems that together with the above demonstrated hydrophobic effect and the higher effective molarity account for the observed substrate activation.

## Discussion

We have constructed a mesoscopic and lamellar metal-organic material that was successfully used as a host catalyst for the condensation reactions in water under neutral conditions. Activity displayed appears to emulate enzyme's way of action in natural systems, wherein the hydrophobic pocket and non-covalent interactions account for the extended efficiency. The mesoscopic host material can also shelter a chiral organocatalyst and two reacting components, facilitating asymmetric transformations under more environmentally friendly conditions, meaning lower catalyst loading, room-temperature conditions and water as a green solvent. The mesoscopic phase of the solid allows for easy diffusion of starting materials and products resulting in procedures promoted by a catalytic amount of the solid. Although the formed lipophilic products prefer the hydrophobic environment in the catalyst system over the aqueous solvent, the obtained products are easily extracted and the solid could be recycled and reused. Furthermore, we have shown that STD NMR is a valuable technique for the *in situ* solution characterization of intermolecular interactions between substrates and dispersed hybrid metal-organic materials, allowing for detection of binding ligands.

## Methods

### Synthesis of the mesoscopic hybrid material Al-ITQ-HB

General experimental information can be found in the Supplementary Methods. Al-ITQ-HB material was synthesized from equimolar quantities of AlCl_3_.6H_2_O (3.1 mmol) and HB acid (3.1 mmol) which were dissolved in two different solutions with 15 ml dimethylformamide in each. The two solutions were mixed and the resulting slurry (pH=2.5) was introduced into a stainless steel autoclave, being heated at 150 °C for 24 h under autogeneous pressure and static conditions. Once cooled to room temperature, the solution (pH=5.5) was filtered and the collected powder was washed with distilled water. Then, the sample was stirred in methanol for 24 h to efficiently remove the remaining unreacted linker and dimethylformamide solvent molecules. Finally, the material was isolated and dried under vacuum at room temperature.

### Knoevenagel condensation reaction

General experimental information can be found in the Supplementary Methods. The mesoscopic hybrid material, Al-ITQ-HB (8.0 mg) was placed in a 1-ml glass vessel. Aldehyde **1a** (0.1 mmol, 15.6 mg) and compound **2** (0.1 mmol, 14.4 mg) were then added. Chloroform (100 μl) and water (100 μl) were subsequently added and the reaction mixture was left to stir vigorously at room temperature for 10 h. The product was extracted with EtOAc (3 × 1 ml), and the solvent evaporated *in vacuo* to give **3a** (Supplementary Methods) in 91% yield as determined by analysis by ^1^H NMR.

### Multicomponent reaction

The mesoscopic hybrid material, Al-ITQ-HB (5.3 mg, 10 mol%) or the corresponding additive (as indicated in the Supplementary Figs 15–17) was placed in a 1-ml glass vessel. Isatin (29.4 mg, 0.2 mmol), dimedone (28 mg, 0.2 mmol) and Ph_3_CH (12.2 mg, 0.05 mmol) were then added. Subsequently, a stock solution of malononitrile in CDCl_3_ was then added (0.6 ml, 0.2 mmol of malononitrile) and the reaction mixture was left to stir vigorously at room temperature. Evolution of the reaction was followed by ^1^H NMR by taking aliquots of the reaction mixture and dissolving the product in d6-DMSO.

### Michael addition of isobutyraldehyde to *trans*-β-nitrostyrene

General experimental information can be found in the Supplementary Methods. The mesoscopic hybrid material, Al-ITQ-HB (24 mg) and organocatalyst **4** (14.1 mg, 0.03 mmol) were placed in a 2-ml glass vessel. *trans*-β-Nitrostyrene (45.0 mg, 0.3 mmol) was then added. Toluene (150 μl) and water (450 μl) were subsequently added followed by isobutyraldehyde (81 μl, 0.9 mmol) and the reaction mixture was left to stir vigorously at room temperature for 24 h. The product was extracted with EtOAc (5 × 1 ml), and host catalyst separated by centrifugation. Volatile components were then removed under reduced pressure and the crude product was purified by column chromatography using hexane/ethyl acetate (90/10) as eluent to yield addition product (Supplementary Fig. 28 and Supplementary Methods) in 96% yield (63.7 mg, 0.29 mmol). Enantiomeric excess was determined to be 98% using HPLC on a chiral stationary phase. (Kromasil 5-Cellucoat, *n*-hexane/isopropanol=70:30, 1 ml min^−1^, 220 nm, t (major)=10.1 min and t (minor)=13.6 min (Supplementary Fig. 31).

### Michael addition of dimethylmalonate to 4-phenyl-3-buten-2-one

The mesoscopic hybrid material, Al-ITQ-HB (24 mg, 0.09 mmol, 30 mol%) and organocatalyst **4** (14.1 mg, 0.03 mmol) were placed in a 2-ml glass vessel. 4-Phenyl-3-buten-2-one (43.9 mg, 0.3 mmol) was then added. Toluene (150 μl) and water (300 μl) were subsequently added followed by dimethylmalonate (69 μl, 0.6 mmol) and the reaction mixture was left to stir vigorously at room temperature for 24 h. The product was extracted with EtOAc (5 × 1 ml), and host catalyst separated by centrifugation. Volatile components were then removed under reduced pressure and the crude product was purified by column chromatography using hexane/ethyl acetate as eluent to yield addition product (Supplementary Fig. 30 and Supplementary Methods) in 98% yield (81.8 mg, 0.29 mmol). Enantiomeric excess was determined to be 96% using HPLC on a chiral stationary phase. (Kromasil 5-amycoat, *n*-hexane/isopropanol=90:10, 1 ml min^−1^, 220 nm, t (minor)=9.79 min and t (mayor)=11.3 min (Supplementary Fig. 33).

### Michael addition of nitromethane to 4-phenyl-3-buten-2-one

The mesoscopic hybrid material, Al-ITQ-HB (24 mg, 0.09 mmol, 30 mol%) and organocatalyst **4** (14.1 mg, 0.03 mmol) were placed in a 2-ml glass vessel. 4-Phenyl-3-buten-2-one (43.9 mg, 0.3 mmol) was then added. Toluene (150 μl) and water (300 μl) were subsequently added followed by nitromethane (150 μl) and the reaction mixture was left to stir vigorously at room temperature for 24 h. The product was extracted with EtOAc (5 × 1 ml), and host catalyst separated by centrifugation. Volatile components were then removed under reduced pressure and the crude product was purified by column chromatography using hexane/ethyl acetate as eluent to yield addition product (Supplementary Fig. 29 and Supplementary Methods) in 87% yield (54.1 mg, 0.26 mmol). Enantiomeric excess was determined to be 92% using HPLC on a chiral stationary phase. (Daicel Chiralcel OJ, *n*-hexane/isopropanol=40:60, 1.5 ml min^−1^, 220 nm, t (minor)=9.3 min and t (mayor)=13.3 min (Supplementary Fig. 32).

## Additional information

**How to cite this article:** García-García, P. *et al*. Organic–inorganic supramolecular solid catalyst boosts organic reactions in water. *Nat. Commun.* 7:10835 doi: 10.1038/ncomms10835 (2016).

## Supplementary Material

Supplementary Information Supplementary figures 1-33, Supplementary Tables 1-5, Supplementary Methods and Supplementary References

## Figures and Tables

**Table 1:**
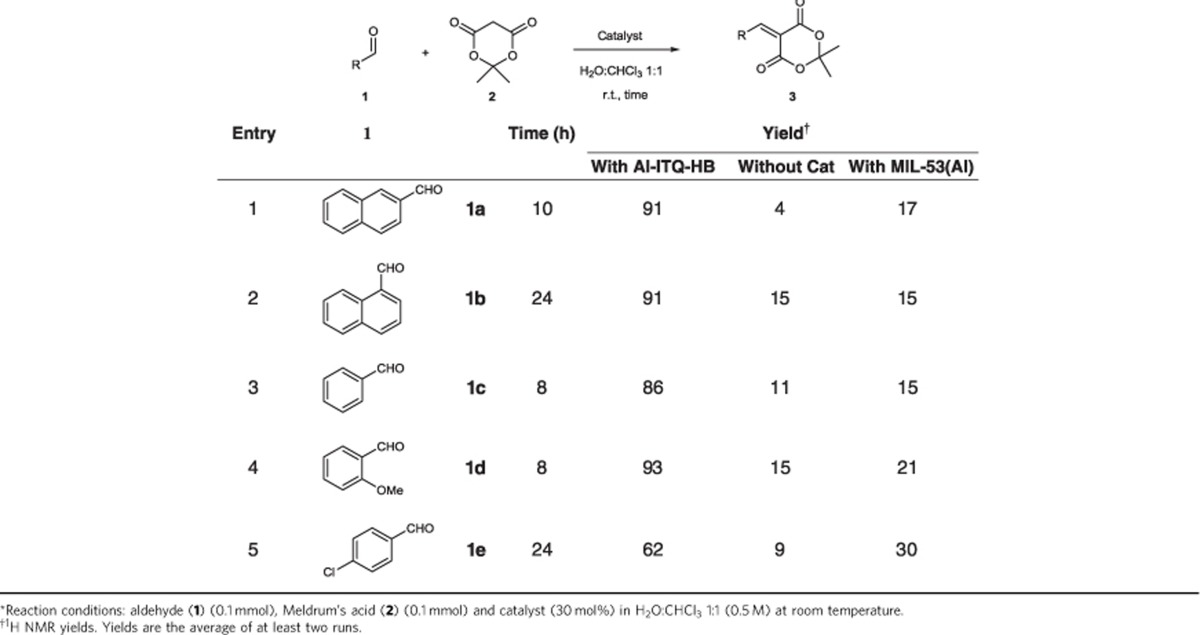
Al-ITQ-HB catalysed Knoevenagel condensation of aldehydes **1** with Meldrum's acid 2*.
