# Supplementary material for: Organic–inorganic supramolecular solid catalyst boosts organic reactions in water
Source: Nat Commun. 2016 Feb 25;7:10835. doi: 10.1038/ncomms10835 (PMC4773429; doi:10.1038/ncomms10835)
Supplement: Supplementary Information — Supplementary figures 1-33, Supplementary Tables 1-5, Supplementary Methods and Supplementary References [file ncomms10835-s1.pdf]

Supplementary Figures

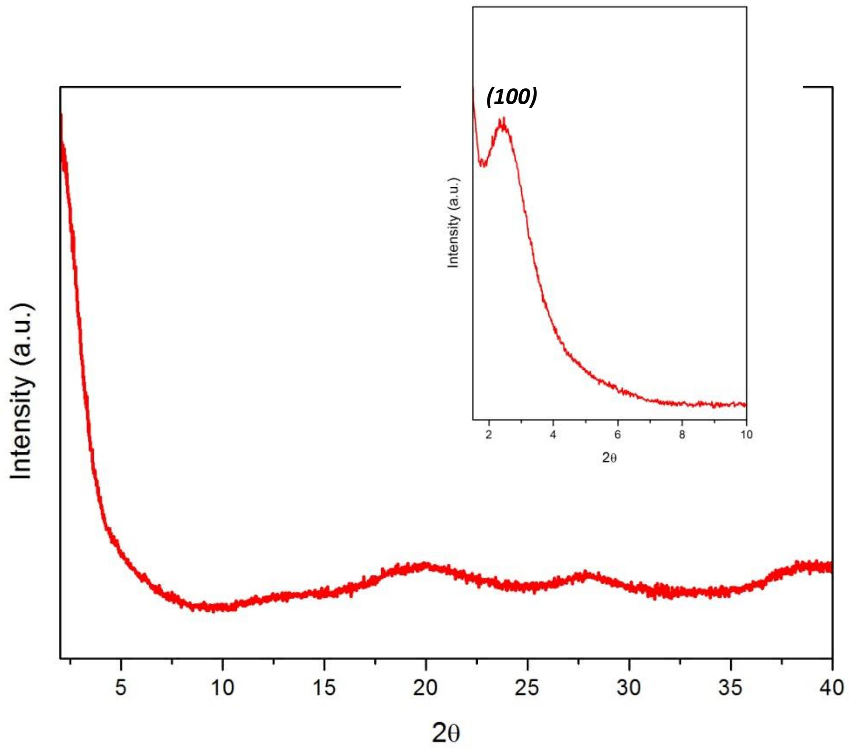

Supplementary Figure 1. XRD pattern of mesoscopic hybrid material, Al-ITQ-HB. In the inset, low-angle XRD diffractogram of Al-ITQ-HB sample is shown.

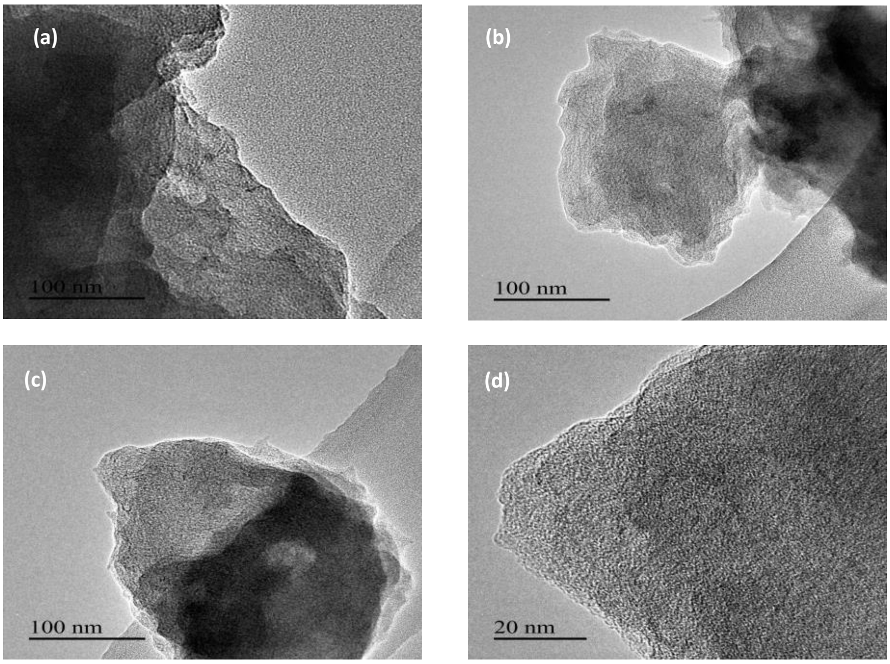

Supplementary Figure 2. HRTEM images of Al-ITQ-HB sample. Scale bars are included in each micrograph.

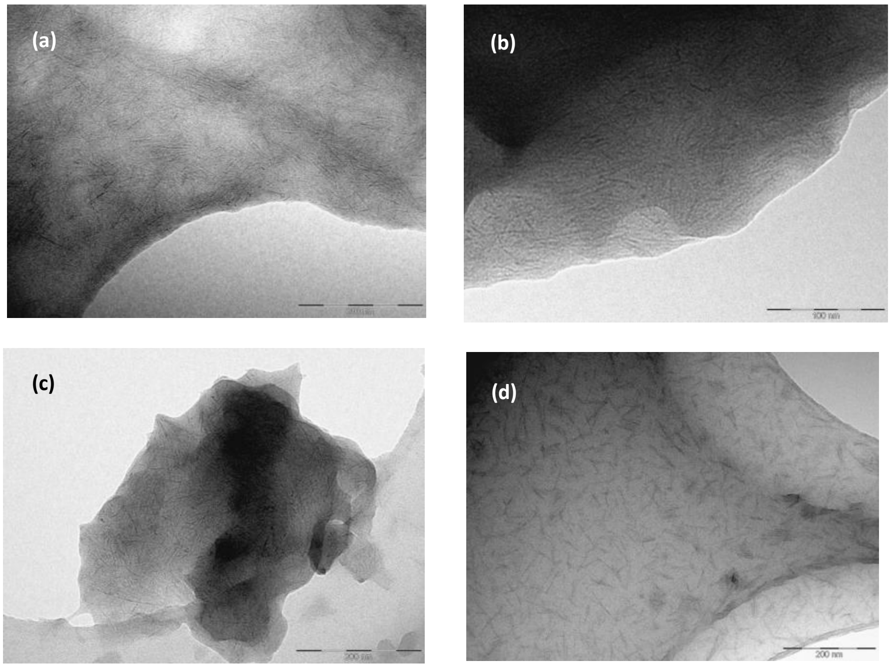

Supplementary Figure 3. TEM images of stable solution derived of post-synthesis treatment of Al-ITQ-HB sample with dichloromethane. Scale bars are included in each micrograph.

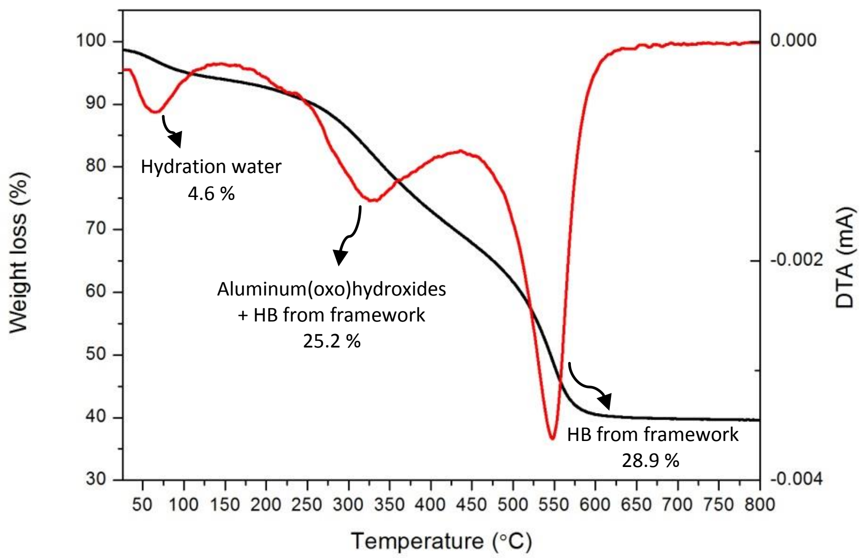

Supplementary Figure 4. TGA and DTA curves of Al-ITQ-HB sample. It is shown the weight loss and the corresponding derivative (TGA and DTA curves) with temperature for the mesoscopic metalorganic hybrid material which allowed establishing, not only the amount of organic spacers incorporated in the Al-ITQ-HB solid, but also their hydrothermal stability. Specifically, and after elimination of the major part of hydration water and residual DMF used as solvent in the synthesis processes (both detected at around 80-150ºC), it was possible to see a main weight loss, between 450ºC and 600ºC, assigned to the presence of *para*-alkyl benzene monocarboxylate molecules (HB) used as spacers. It is important to remark the weight loss observed between 250ºC and 400ºC assigned to the decomposition (dehydration phenomenon) of $AlO_4(OH)_2$ units present in the 1D inorganic chains,[1] which would confirm again the presence of assembled lamellar organic-inorganic sub-domains integrated into the mesoscopic framework. This latter weight loss associated with the oxygen elimination present in the $[AlO_6]$ octahedral groups would explain the higher organic content detected from TGA than from CHNS analysis (Supplementary Table 1). However, in this temperatures range, contribution of alkyl chains from incorporated organic linkers (HB) should also be considered. Thus, the organic content calculated from the thermogravimetric curves and CHNS elemental analysis (Supplementary Table 1) confirmed the presence of the organic linker compounds stabilized into the network of mesoscopic Al-ITQ-HB solid.

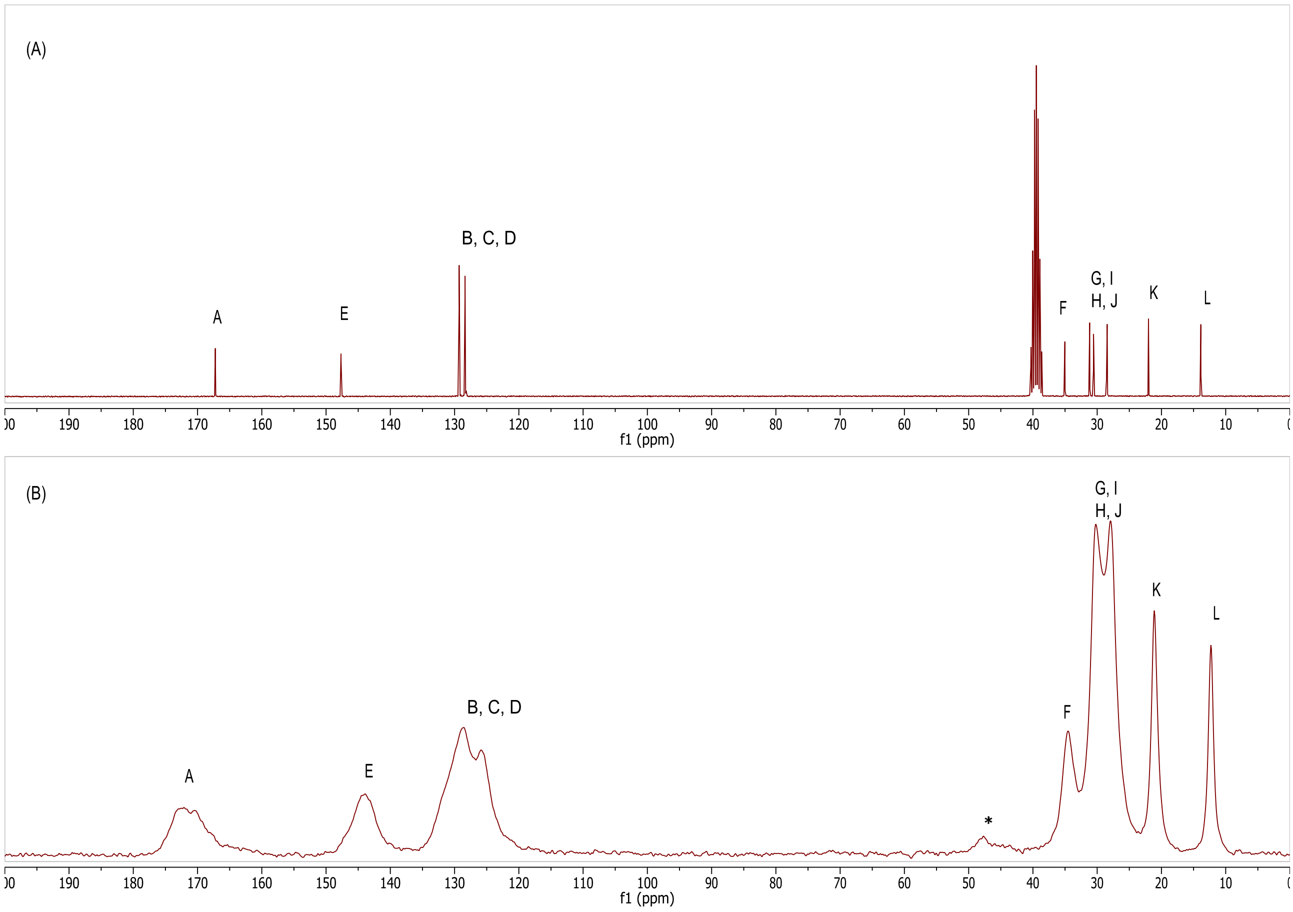

Supplementary Figure 5. (A) ^{13}C NMR spectrum (in d6-DMSO) of pure organic compound HB used as linker to synthesize Al-ITQ-HB. **(B)** ^{13}C CP/MAS NMR spectrum of Al-ITQ-HB. Asterisks represent spinning bands.

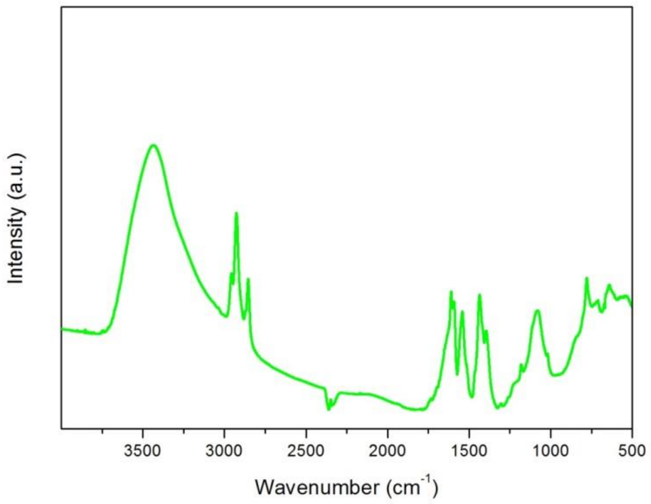

Supplementary Figure 6. FTIR spectrum of Al-ITQ-HB. The FTIR spectrum showed the stretching vibration mode of the hydroxyl group (-OH) being located between 3600 and 3200 cm^{-1} associated to hydration water and aluminum oxo-hydroxide ($AlO_4(OH)_2$) structural units. Moreover, three typical bands were also observed due to $–CH_2–$ groups from the long tails of the HB spacer at 2950, 2920 and 2850 cm^{-1}. The signals located at 1590 and 1490 cm^{-1} were assigned to asymmetric stretching vibrations of ring aromatic groups and the bands from carboxylate groups were detected at 1610 and 1440 cm^{-1}. In conclusion, IR spectroscopy verified the presence of the organic spacers in the walls of mesoscopic hybrid materials, confirming the NMR results.

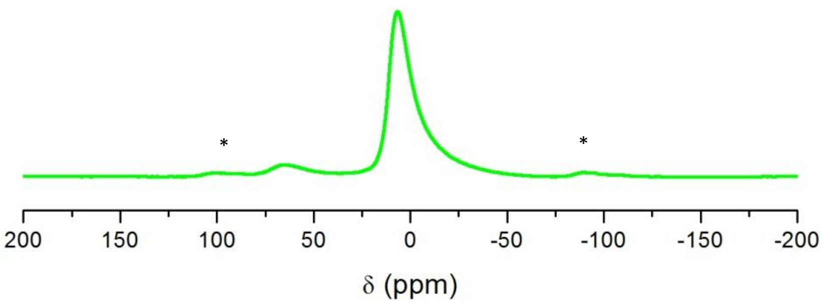

Supplementary Figure 7. ^{27}Al BD/MAS NMR spectra of Al-ITQ-HB. Asterisks represent spinning bands.

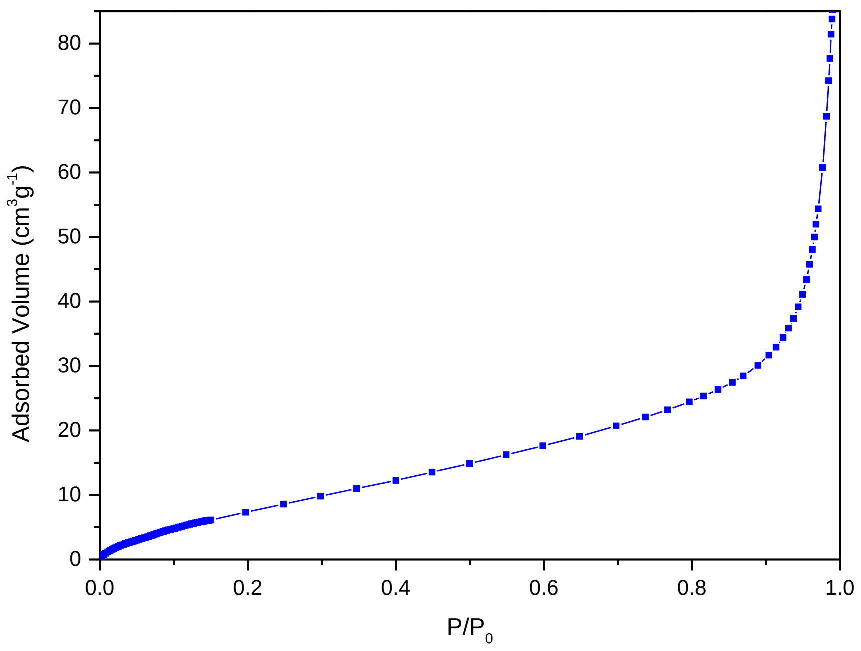

Supplementary Figure 8. Ar adsorption isotherm of Al-ITQ-HB sample. Ar adsorption isotherm was performed at 87.3 K in an ASAP 2010 apparatus from Micromeritics, after pretreating the samples under vacuum at 333 K overnight. Textural properties were analyzed from Argon adsorption isotherm for Al-ITQ-HB solid, being estimated a reduced surface area and porous volume (S_{BET} ~35 m^2g^{-1}, V_{TOTAL} ~0.11 cm^3g^{-1}) due to, probably, the big amount of organic content (~40% wt) present in the hybrid material that hinders the correct gas adsorption along the mesocavities. The marked hydrophobic character of the material would reinforce this phenomenon.

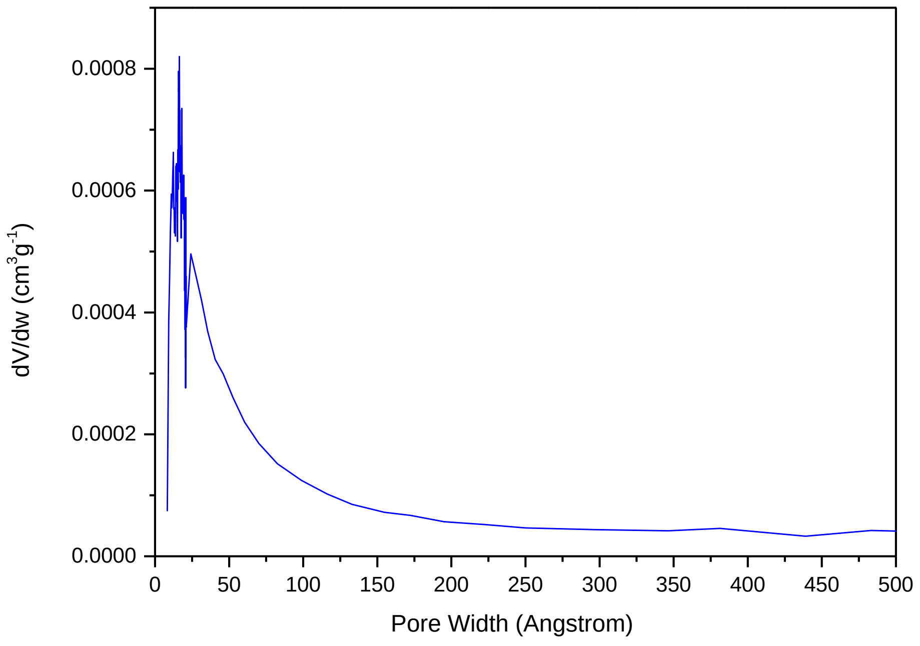

Supplementary Figure 9. Hörvath-Kawazoe pore size distribution obtained from Ar adsorption isotherm of Al-ITQ-HB sample. The Hörvath-Kawazoe pore size distribution showed that the majority of pores were centered at approximately 20-25 Å, being this value coincident with that obtained from electronic microscopy, remarking the mesoscopic nature of the Al-ITQ-HB material.

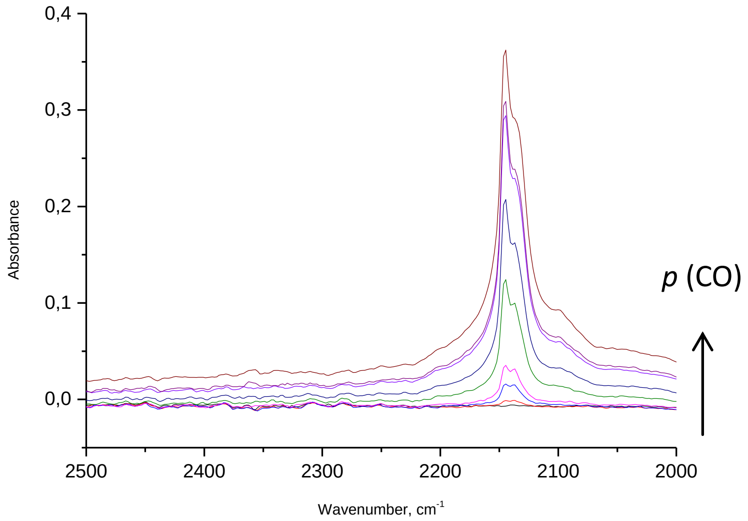

Supplementary Figure 10. Difference FTIR spectra of increasing amounts of CO adsorbed at low temperature (100 K) on MIL-53 (Al) (self-supporting wafer). This technique allowed studying the Lewis acidity and their associated strength in the samples. Prior to CO adsorption the sample was heated in the high vacuum chamber at 423 K to remove adsorbed species. The band observed at 2145 cm^{-1} is indicative of weakly adsorbed CO evidencing the presence of Lewis acidic sites. A complete disappearance of this band is observed at 100K by subsequent treatment of the sample under vacuum.

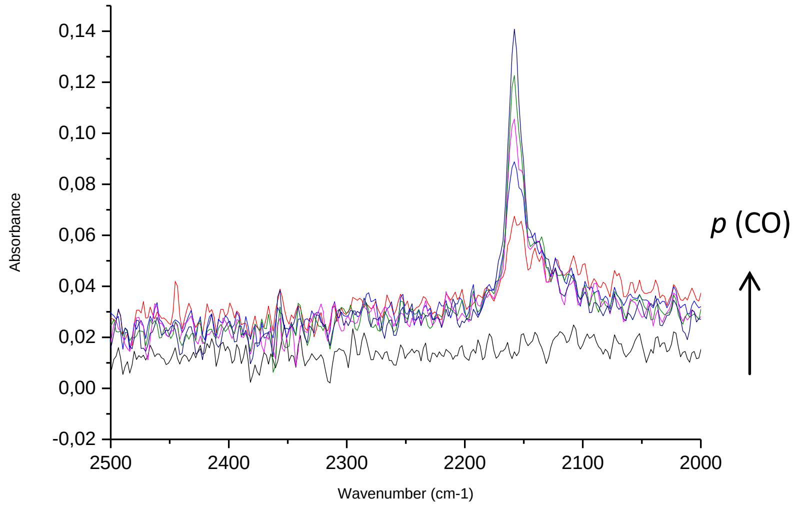

Supplementary Figure 11. Difference FTIR spectra of increasing amounts of CO adsorbed at low temperature (100 K) on Al-ITQ-HB (MOF:SiO_2 1.5:1 wafer). The band observed at 2158 cm^{-1} is indicative of weakly adsorbed CO evidencing the presence of Lewis acidic sites. A complete disappearance of this band is observed at 100K by subsequent treatment of the sample under vacuum.

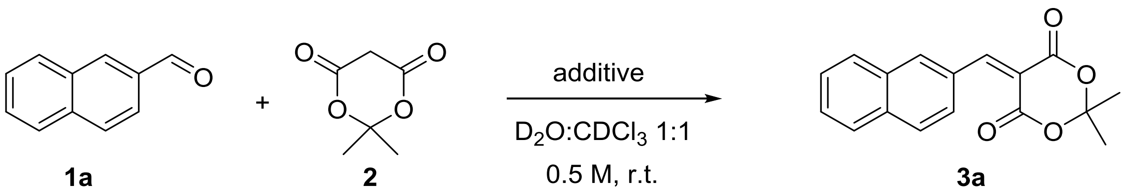

Additive	Initial reaction rate[a]
Al-ITQ-HB	162
p-heptylbenzoic acid (HB)	3
sodium p-heptylbenzoate (Na HB)	3
MIL-53 (Al)	0.8
No additive	0.3

[a]moles of product formed per hour

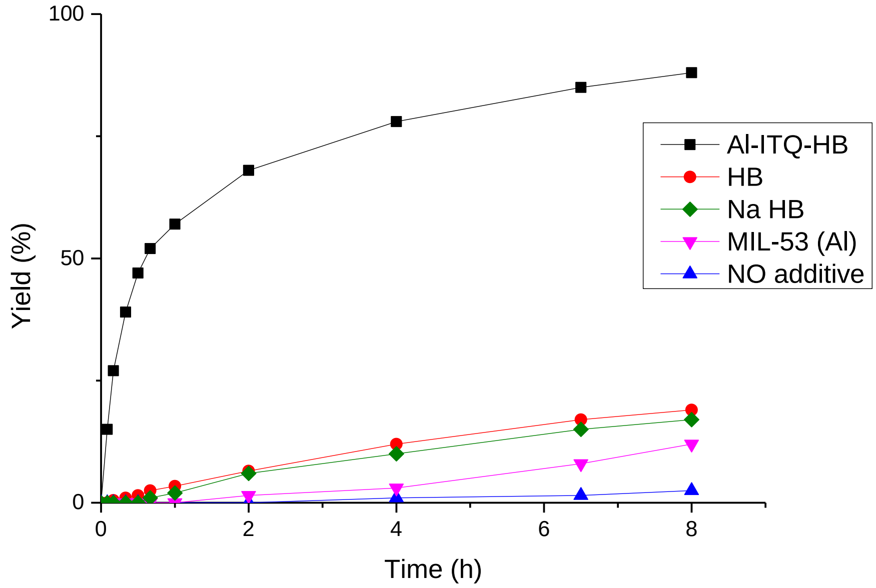

Supplementary Figure 12. Kinetic study of the Knoevenagel condensation reaction in the presence of D_2O. It is shown the % yield of the Knoevenagel product **3a** over time when using different additives (as shown in the legend and in the table) in the condensation reaction in the presence of D_2O. Experiments were carried out with 30 mol% of additive and yield determined by analysis by ^1H NMR of aliquots taken from the reaction mixture at different times as shown in the figure. An important increase in the reaction rate can be observed in the case of Al-ITQ-HB versus utilization of 4-heptylbenzoic acid, sodium 4-heptylbenzoate, MIL-53 (Al) or no catalyst at all.

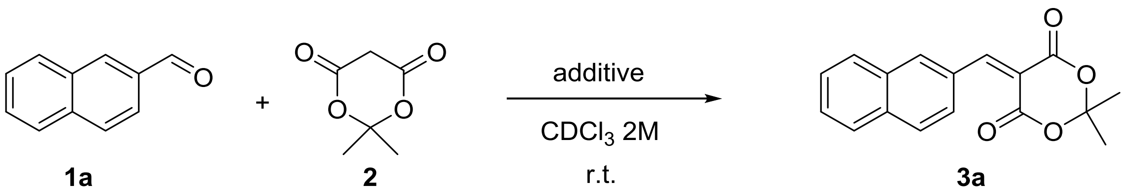

Additive	Initial reaction rate[a]
Al-ITQ-HB	84
p-heptylbenzoic acid (HB)	4
MIL-53 (Al)	2.5
No additive	0.8

[a]moles of product formed per hour

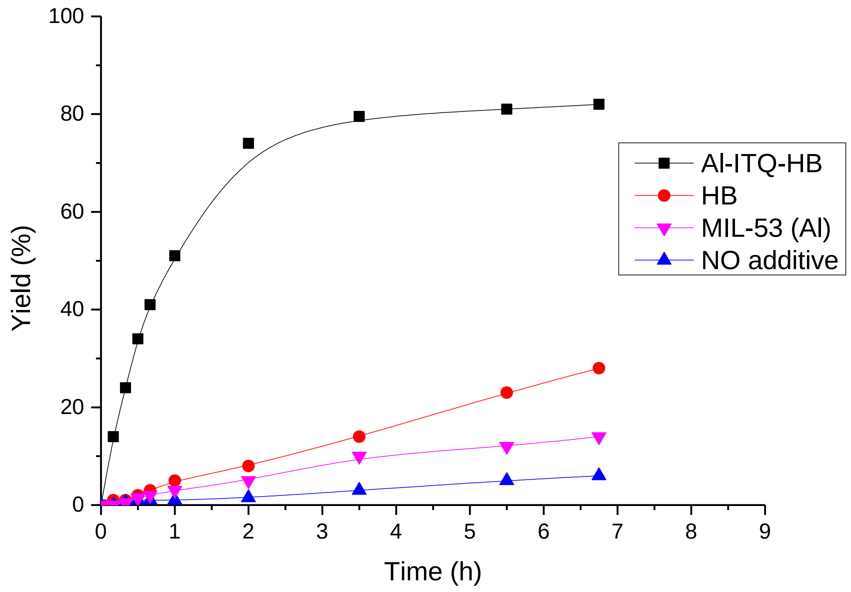

Supplementary Figure 13. Kinetic study of the Knoevenagel condensation reaction in organic solvent: $CDCl_3$. It is shown the % yield of the Knoevenagel product **3a** over time when using different additives (as shown in the legend and in the table) in the condensation reaction carried out in an organic solvent such as $CDCl_3$. An important increase in the reaction rate can be observed in the case of Al-ITQ-HB versus utilization of 4-heptylbenzoic acid, MIL-53 (Al) or no catalyst at all.

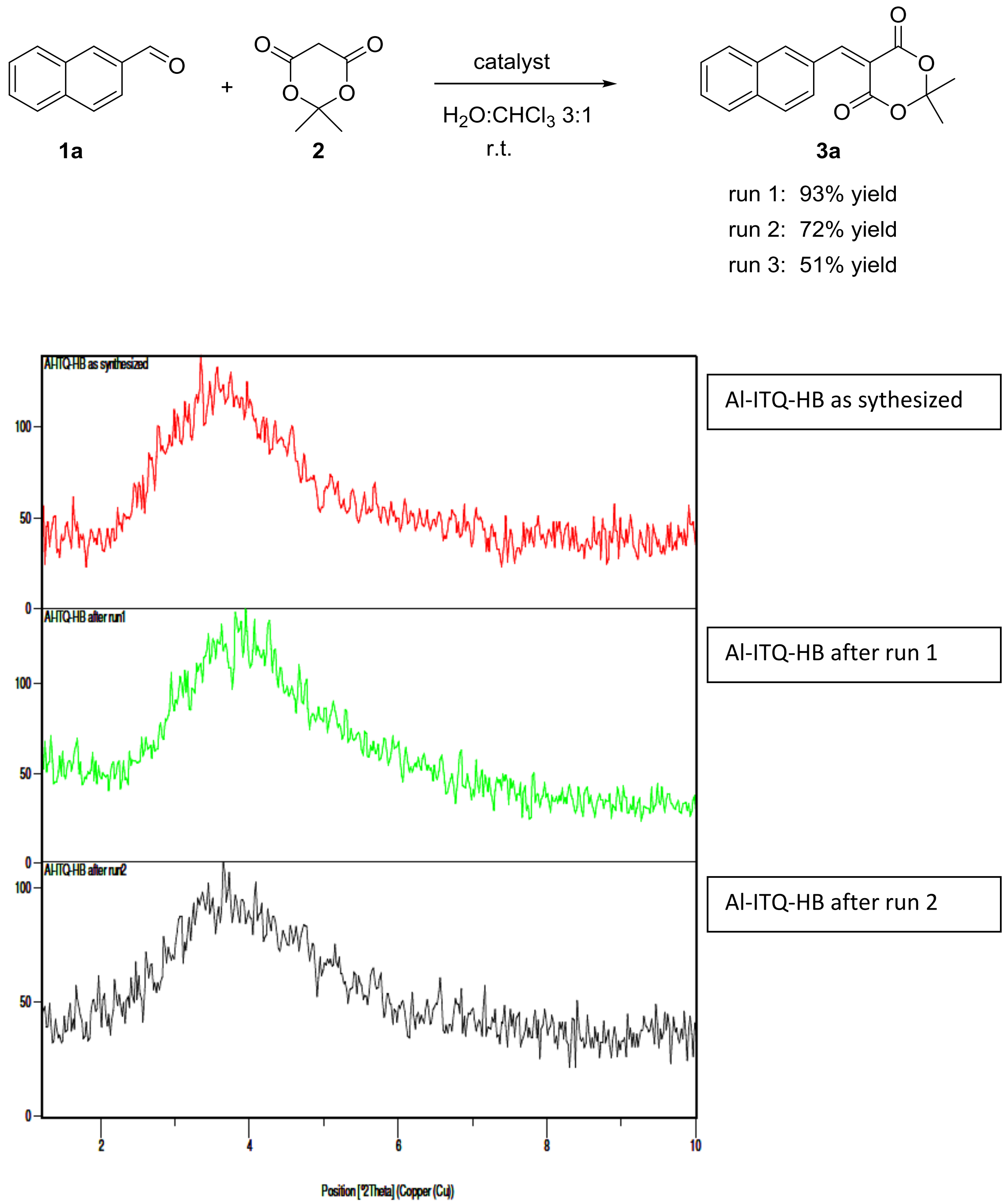

Supplementary Figure 14. Catalyst reuse in the Knoevenagel condensation of aldehyde 1a with Meldrum's Acid 2. It is shown the low-angle XRD pattern of mesoscopic hybrid material, Al-ITQ-HB as synthesized, and after uses 1 and 2. The attained **3a** yields after each run are also shown. Reaction conditions: the mesoscopic hybrid material, Al-ITQ-HB (5.3 mg, 0.02 mmol, 20 mol%) was placed in a 1 mL glass vessel. Aldehyde **1a** (0.1 mmol, 15.6 mg) and compound **2** (0.1 mmol, 14.4 mg) were then added. Chloroform (50 µL) and water (150 µL) were subsequently added and the reaction mixture was left to stir vigorously at room temperature for 6 h. The product was

extracted with EtOAc (3 x 1 mL), and solid separated by centrifugation. The solvent was evaporated *in vacuo* to give condensation product **3a** in 93% yield as determined by analysis by ^{1}H NMR using triphenylmethane as internal standard. The mesoscopic hybrid material, Al-ITQ-HB was then dried at 70ºC for 2 hours before submitting it to the next run. The resulting recovered catalyst was weighed and the next reaction was run using amounts of solvent and reactant proportional to the recovered catalyst amount to maintain the same substrate/catalyst ratio and substrate concentration.

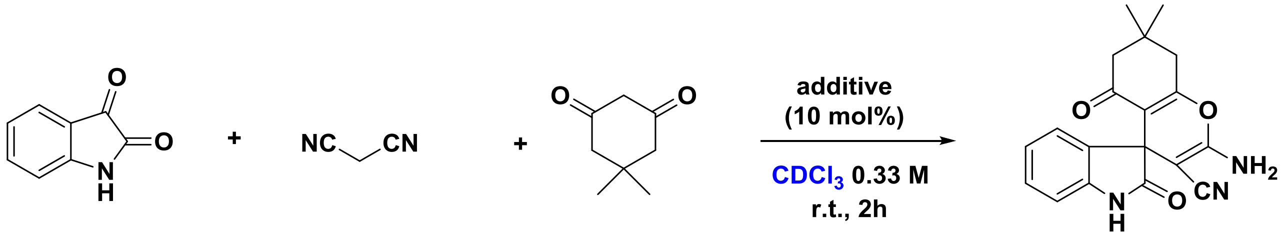

additive	Initial Rate	Yield (%)
Al-ITQ-HB	2.3	91
MIL-53 (Al)	--	10
HB (2.5 mol%)	--	--[a]
HB (10 mol%)	--	--[a]
---	--	--[a]

[a]desired product not detected by ^{1}H NMR

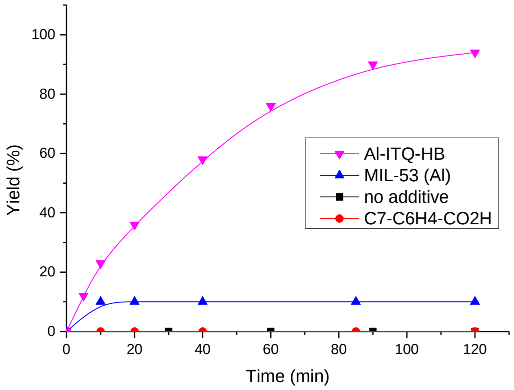

Supplementary Figure 15. . Kinetic study of the multicomponent reaction of isatin, dimedone and malononitrile in organic solvent: $CDCl_3$. It is shown the % yield of the spirooxiindole product over time when using different additives (as denoted in the Table and in the legend) in the multicomponent reaction carried out in an organic solvent such as $CDCl_3$. Evolution of the reaction was followed by ^{1}H NMR by taking aliquots of the reaction mixture and dissolving the product in d6-DMSO. Ph_3CH was used as internal standard.

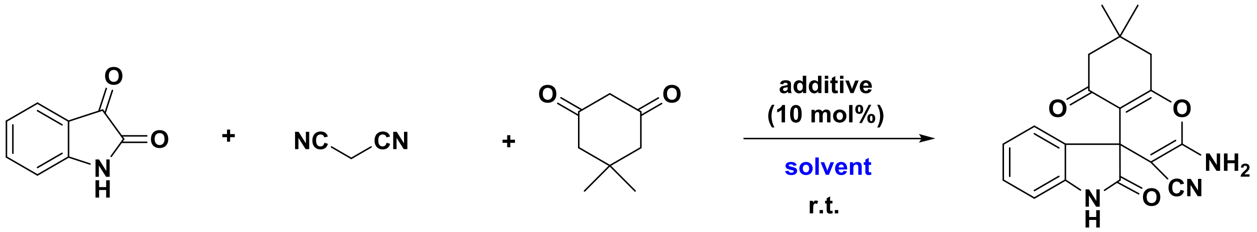

additive	solvent	Initial Rate	Yield (%)/ Time (min)
Al-ITQ-HB	$CDCl_3$ (0.33 M)	2.3	94/120
Na stearate	H_2O (0.33 M)	1.9	85/160
Al-ITQ-HB	$CDCl_3$:H_2O (0.17 M)	3.0	95/ 90
Na stearate	$CDCl_3$:H_2O (0.17 M)	2.6	82/150

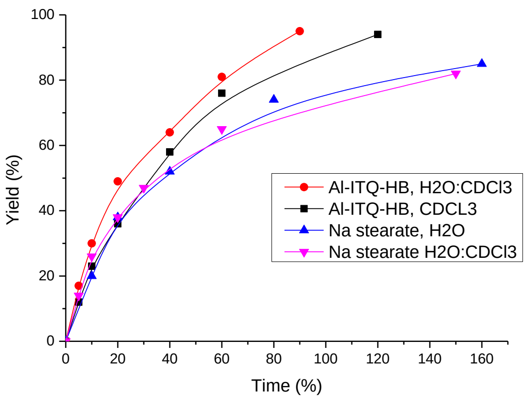

Supplementary Figure 16. Kinetic study of the multicomponent reaction of isatin, dimedone and malononitrile: aqueous micellar catalysis *versus* Al-ITQ-HB catalystis. It is shown the % yield of the spirooxiindole product over time when using Al-ITQ-HB versus sodium stearate (micellar catalysis) in the multicomponent reaction.

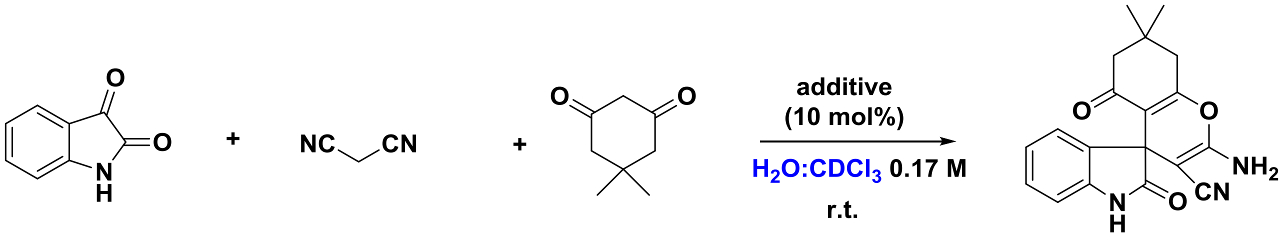

additive	Yield (%)/ Time (min)	Initial Rate
---	42/ 90	0.7
Al-ITQ-HB	95/ 90	3.0
MIL-53 (Al)	45/120	0.4
HB (2.5 mol%)	51/140	0.4
Na stearate	82/150	2.6

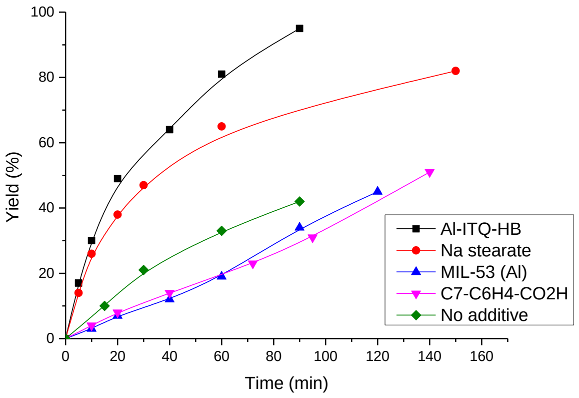

Supplementary Figure 17. Kinetic study of the multicomponent reaction of isatin, dimedone and malononitrile in the presence of H_2O. It is shown the % yield of the spirooxiindole product over time when using different additives (as shown in the Table and in the legend) in the multicomponent reaction carried out in H_2O: $CDCl_3$ as solvent.

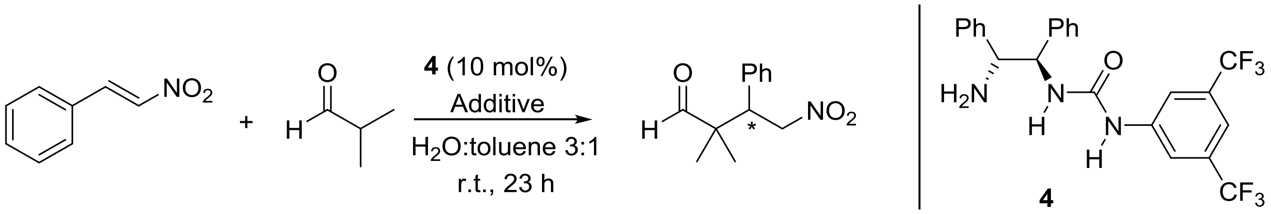

Additive	Initial reaction rate[a]
Al-ITQ-HB	8.9
sodium p-heptylbenzoate (Na HB)	4.4
No additive	0.4
p-heptylbenzoic acid (2.5 mol%)	10

[a]moles of product formed per hour

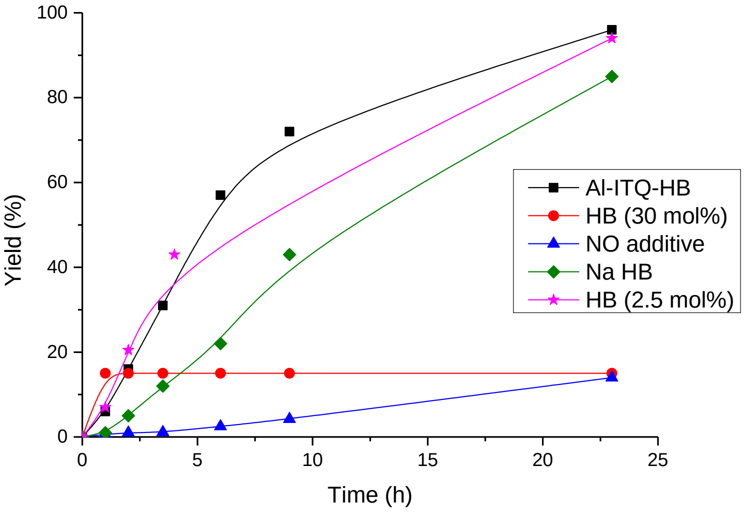

Supplementary Figure 18. Kinetic study of the organocatalytic Michael-type reaction of isobutyraldehyde to *trans*-β-nitrostyrene, in the presence of H_2O. It is shown the % yield of the Michael-type addition product over time when using different additives (as shown in the table and in the legend). Experiments were carried out with 30 mol% of additive (except noted) and yield determined by GC analysis (using dodecane as external standard) of aliquots taken from the reaction mixture at different times as shown in the figure. An important increase in the reaction rate can be observed in the case of Al-ITQ-HB versus utilization of 4-heptylbenzoic acid or no catalyst at all. Rise in reaction rate was also observed under micellar catalytic conditions when using sodium 4-heptylbenzoate, although reaction rate acceleration was more prominent when using our supramolecular host catalyst Al-ITQ-HB.

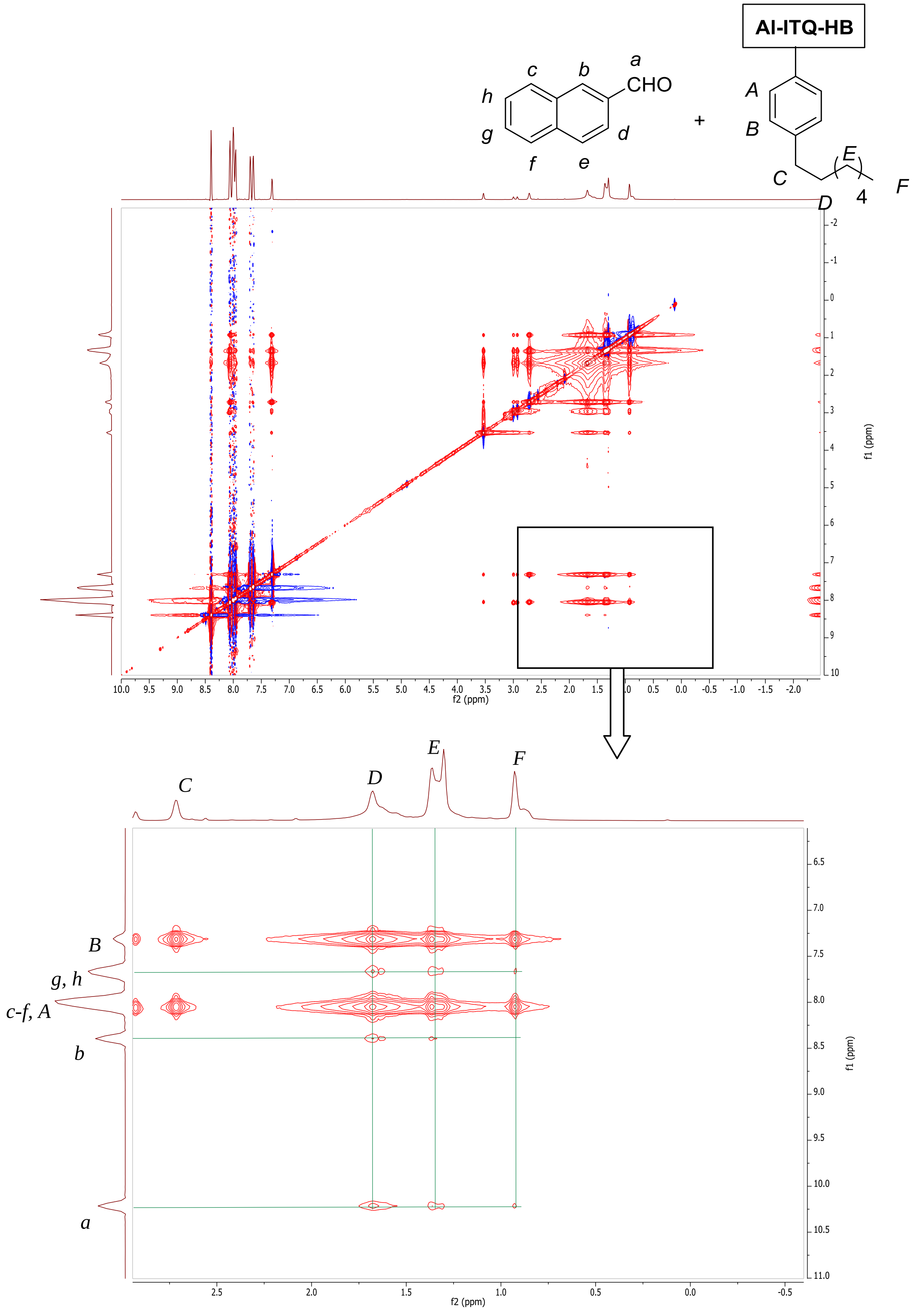

Supplementary Figure 19. 2D NOESY (800 MHz, $CDCl_3$, 50 ms): full spectrum (top) and expansion (bottom) of suspension mixture of naphthaldehyde **1a** and Al-ITQ-HB at 298 K. The expansion (bottom) clearly shows NOE between aromatic protons of the aldehyde and protons in the alkyl groups of Al-ITQ-HB.

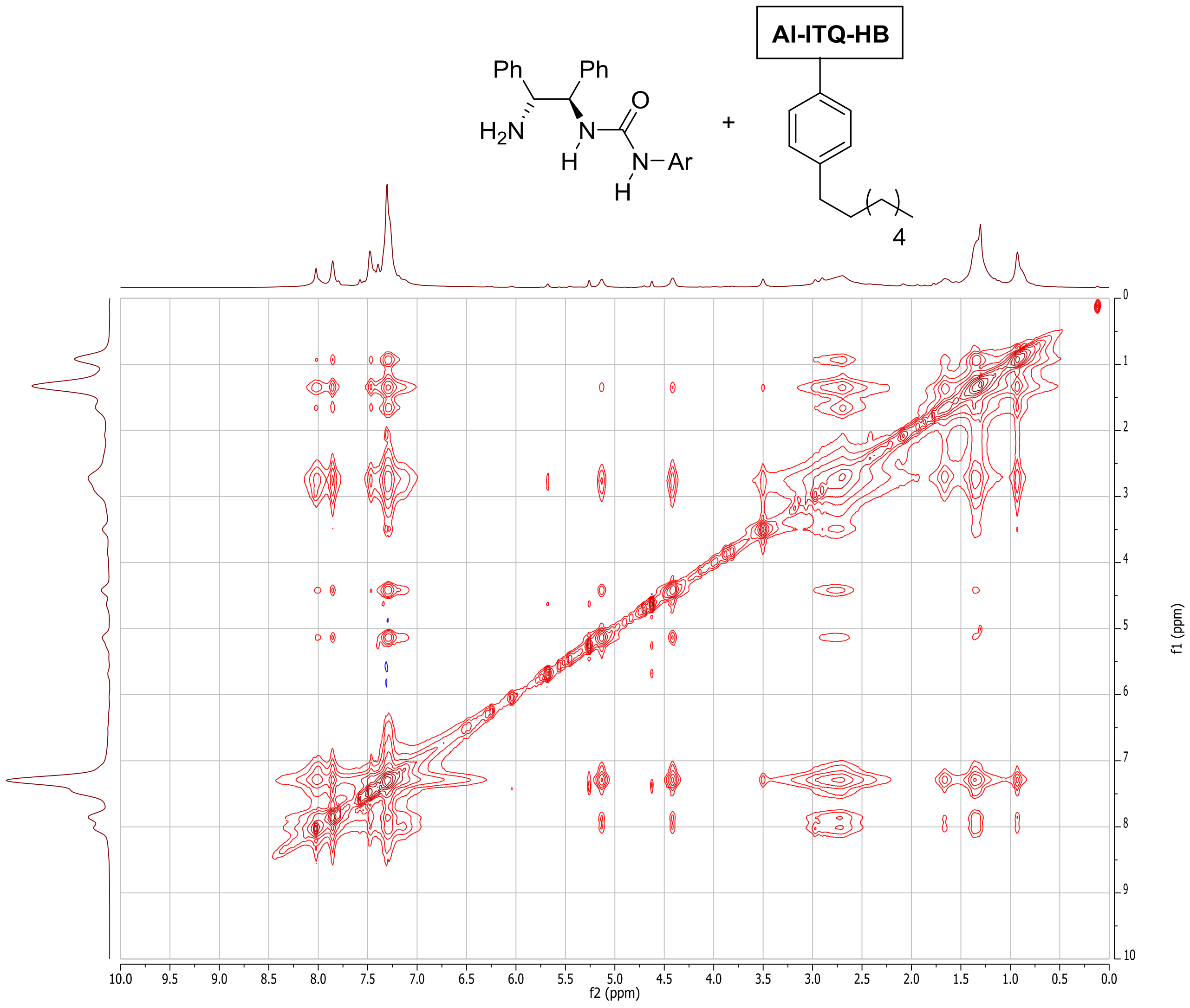

Supplementary Figure 20. 2D NOESY (800 MHz, $CDCl_3$, 50 ms): mixture of organocatalyst **4** and Al-ITQ-HB at 298 K. The extensive NOE observed point out the close contact between organocatalyst **4** and Al-ITQ-HB.

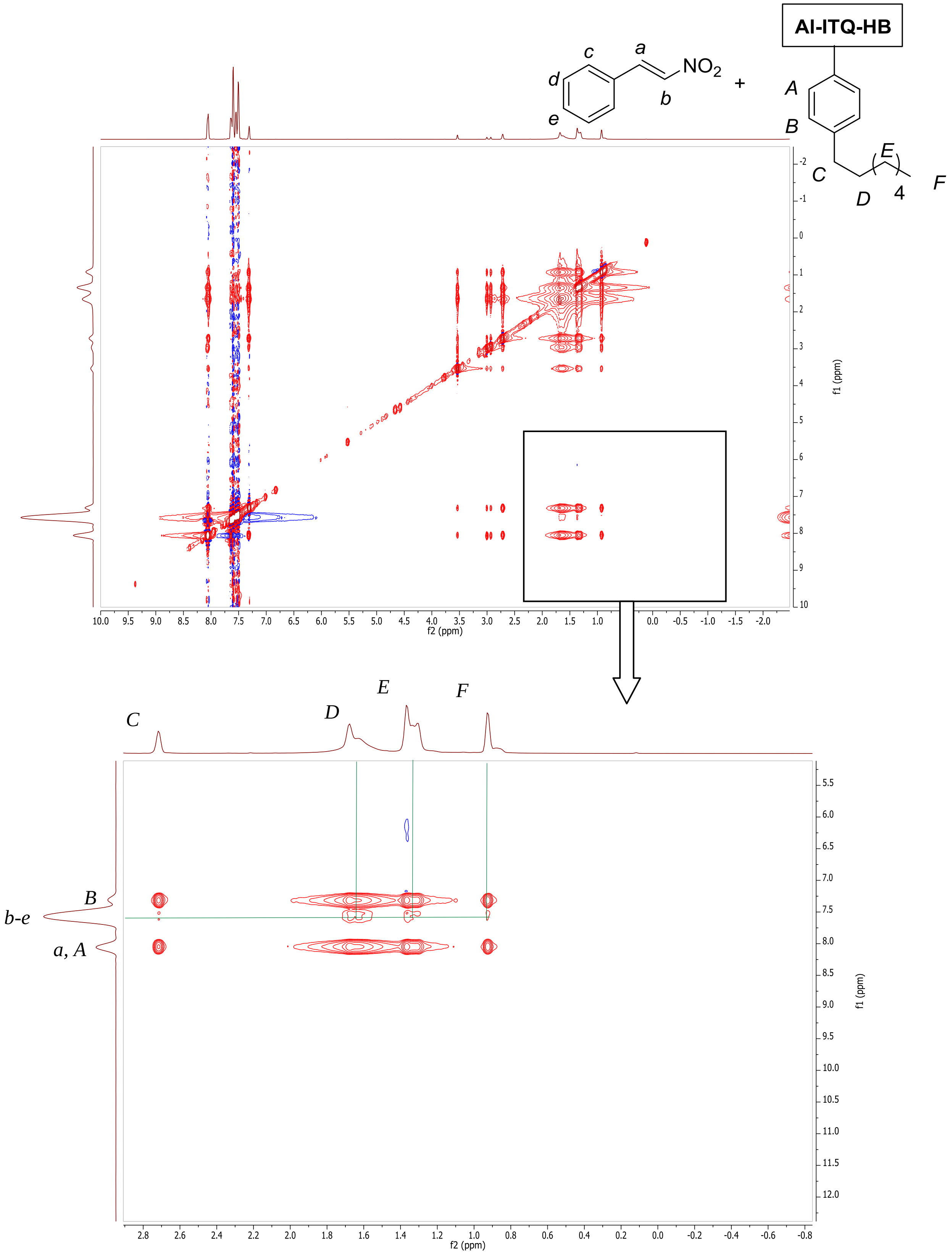

Supplementary Figure 21. 2D NOESY (800 MHz, $CDCl_3$, 150ms): full spectrum (top) and expansion (bottom) of mixture of nitrostyrene and Al-ITQ-HB at 298 K. The expansion (bottom) clearly shows NOE between aromatic protons of the nitrostyrene and protons in the alkyl groups of Al-ITQ-HB.

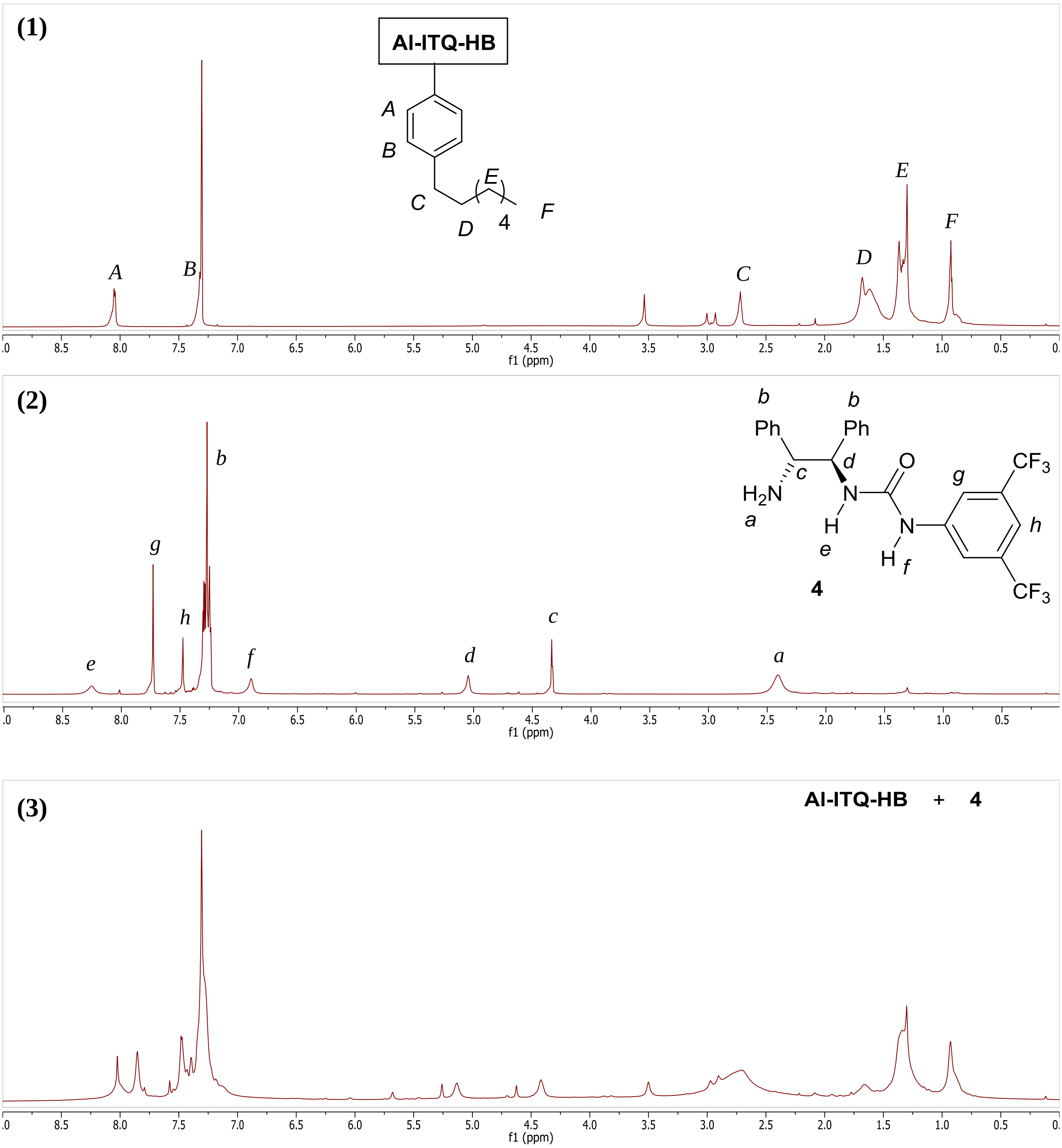

Supplementary Figure 22. ^{1}H NMR (800 MHz, $CDCl_3$) at 298 K of **(1)**: suspension of Al-ITQ-HB in $CDCl_3$, **(2)** Organocatalyst **4** in $CDCl_3$ and (3) organocatalyst **4** and Al-ITQ-HB mixture in $CDCl_3$. Changes in chemical shifts from organocatalyts **4** along with an increase in signal linewidths are clearly observed in the presence of Al-ITQ-HB.

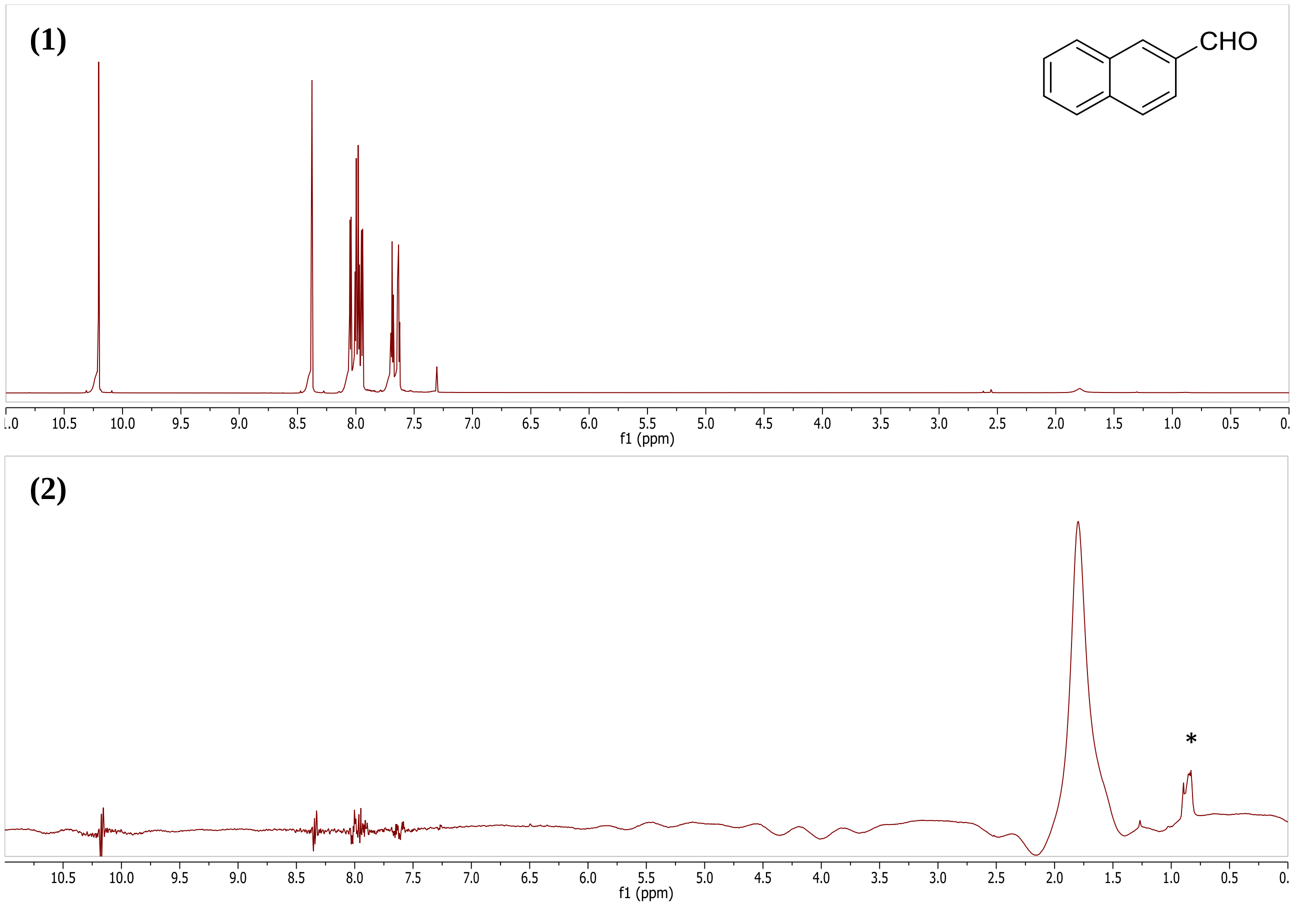

Supplementary Figure 23. STD studies: Blank experiment. (1) ^{1}H NMR reference spectrum of naphthaldehyde **1a**. (2) STD spectrum of the same solution with saturation at 0.9 ppm. Saturation is indicated with an asterisk. Naphthaldehyde resonances doesn’t appear in the STD experiment, only subtraction artifacts are present.

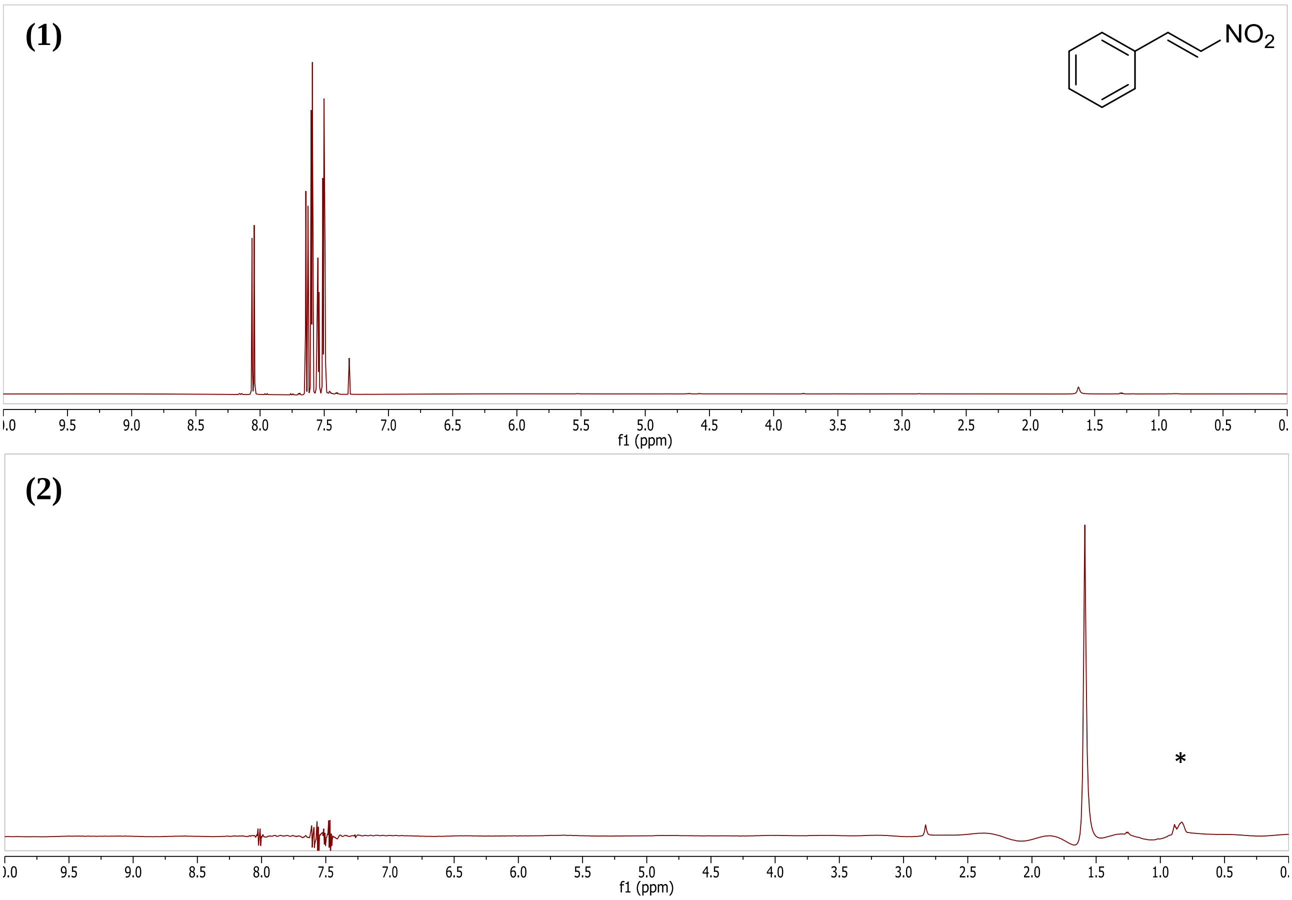

Supplementary Figure 24. STD studies: Blank experiment. (1) ^{1}H NMR reference spectrum of nitrostyrene. (2) STD spectrum of the same solution with saturation at 0.9 ppm. Saturation is indicated with an asterisk. Nitrostyrene resonances doesn't appear in the STD experiment, only subtraction artifacts are present.

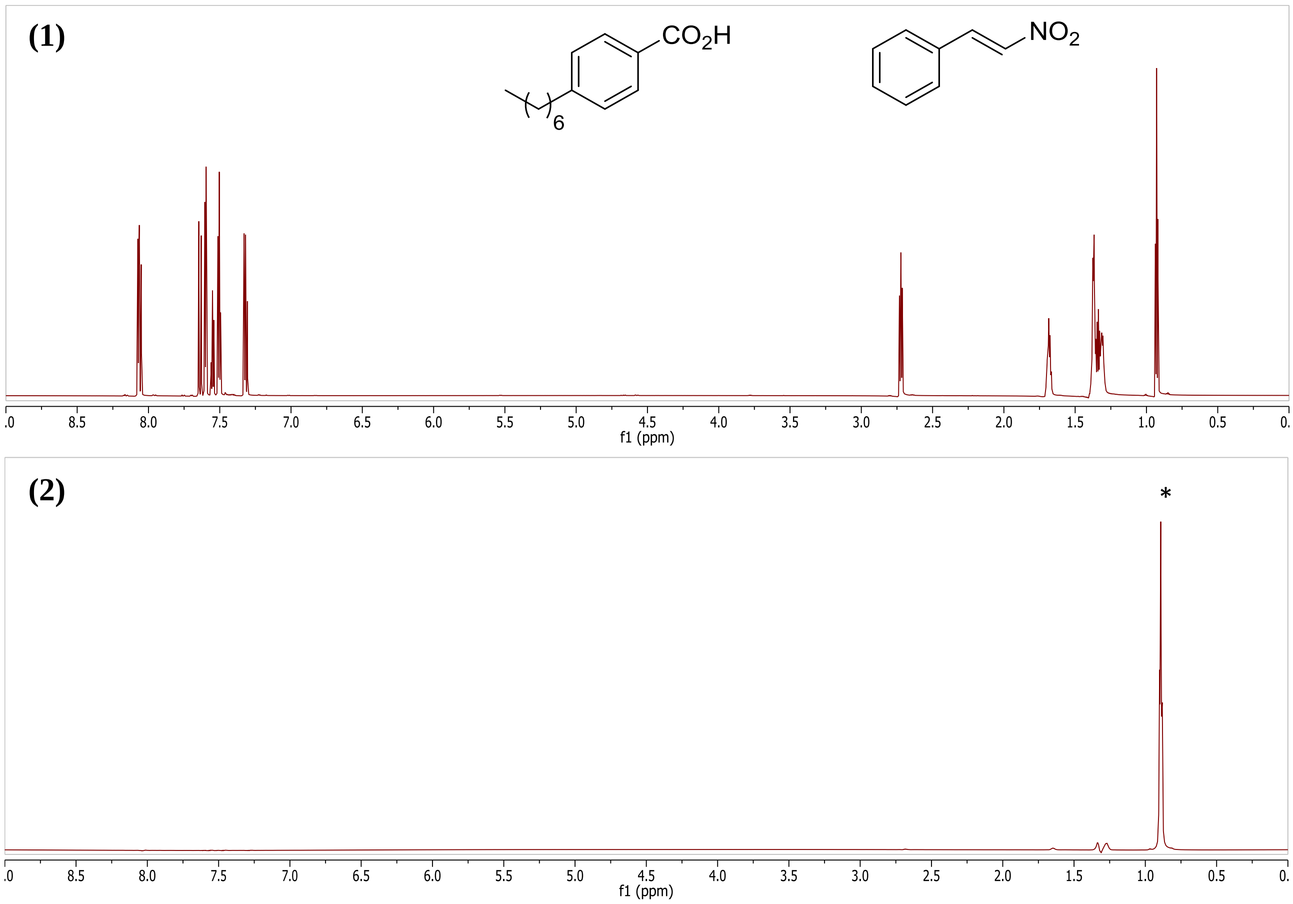

Supplementary Figure 25. STD studies: Blank experiment. (1) ^{1}H NMR reference spectrum of the mixture of nitrostyrene and 4-heptylbenzoic acid (HB). (2) STD spectrum of the same solution with saturation at 0.9 ppm. Saturation is indicated with an asterisk. Nitrostyrene and HB resonances doesn't appear in the STD experiment.

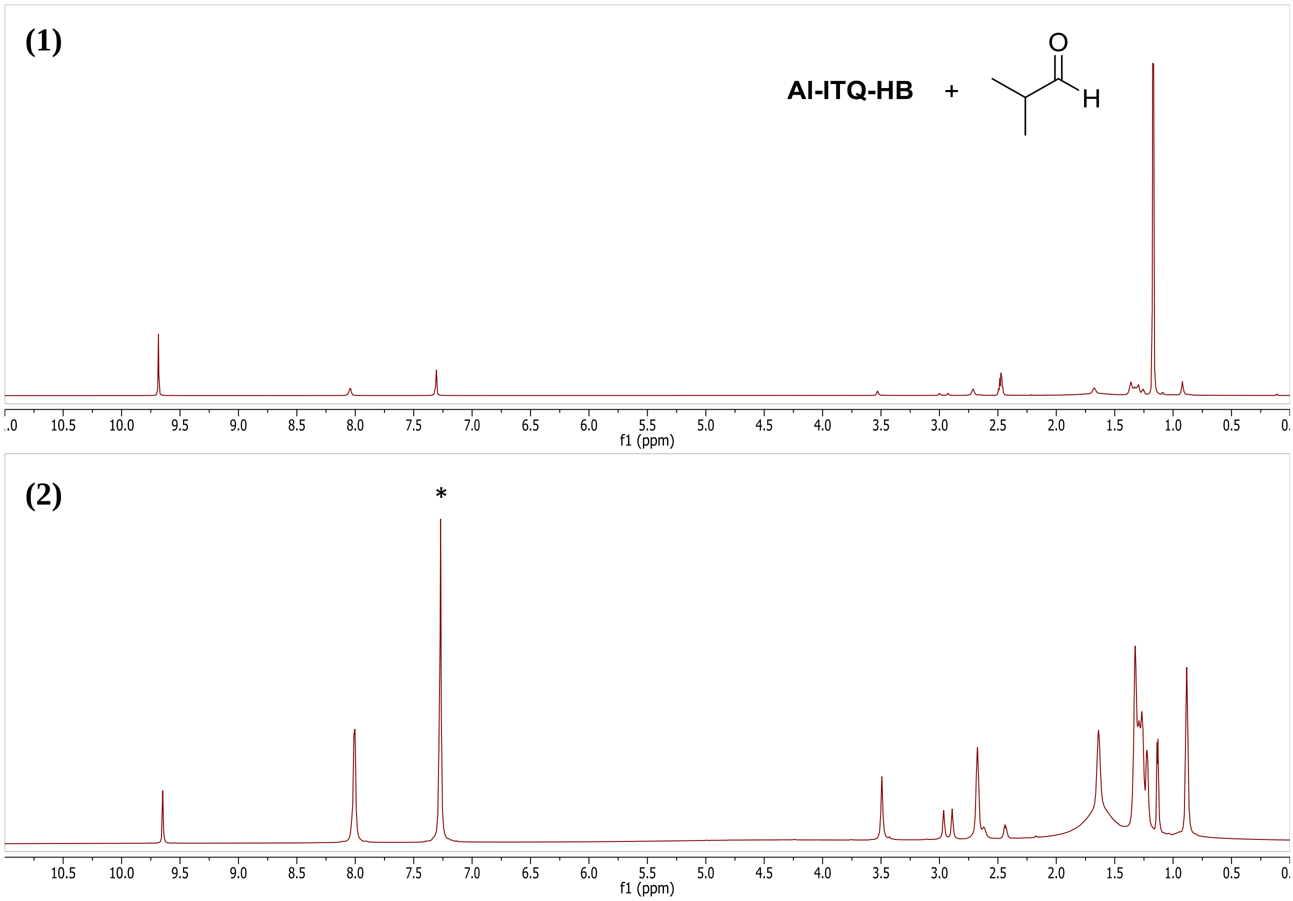

Supplementary Figure 26. STD studies. (1) ^{1}H NMR reference spectrum of isobutyraldehyde and Al-ITQ-HB suspension. (2) STD spectrum of the same suspension with saturation of aromatic signal (7.2 ppm) of Al-ITQ-HB. Signal saturated is indicated with an asterisk. All of the isobutyraldehyde resonances appear in the STD experiment as in the reference ^{1}H NMR demonstrating that isobutyraldehyde is indeed binding to Al-ITQ-HB.

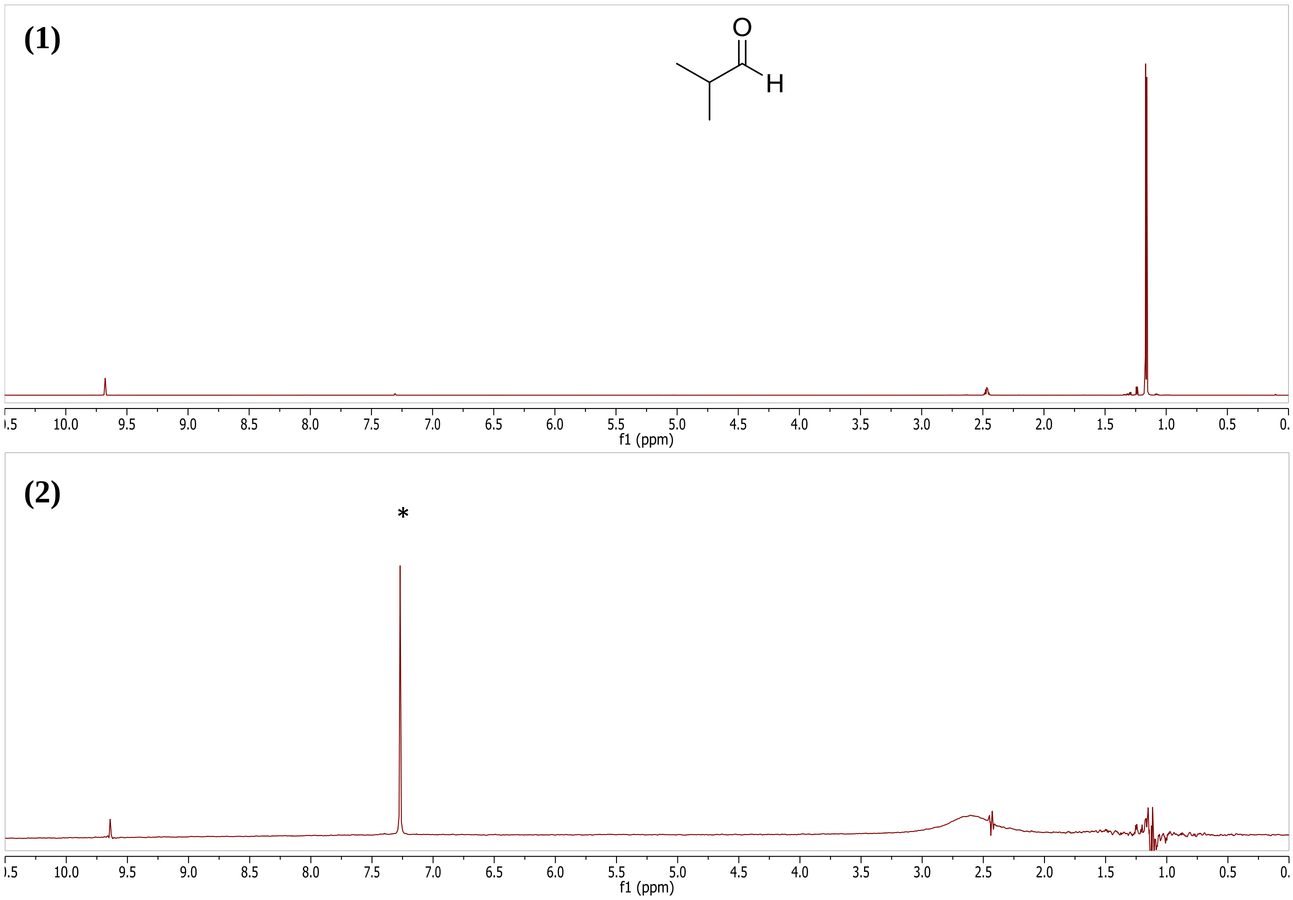

Supplementary Figure 27. STD studies: Blank experiment. (1)^{1}H NMR reference spectrum of isobutyraldehyde. (2) STD spectrum of the same solution with saturation at 7.2 ppm. Saturation is indicated with an asterisk. Most of the isobutyraldehyde resonances doesn’t appear in the STD experiment and subtraction artifacts are present.

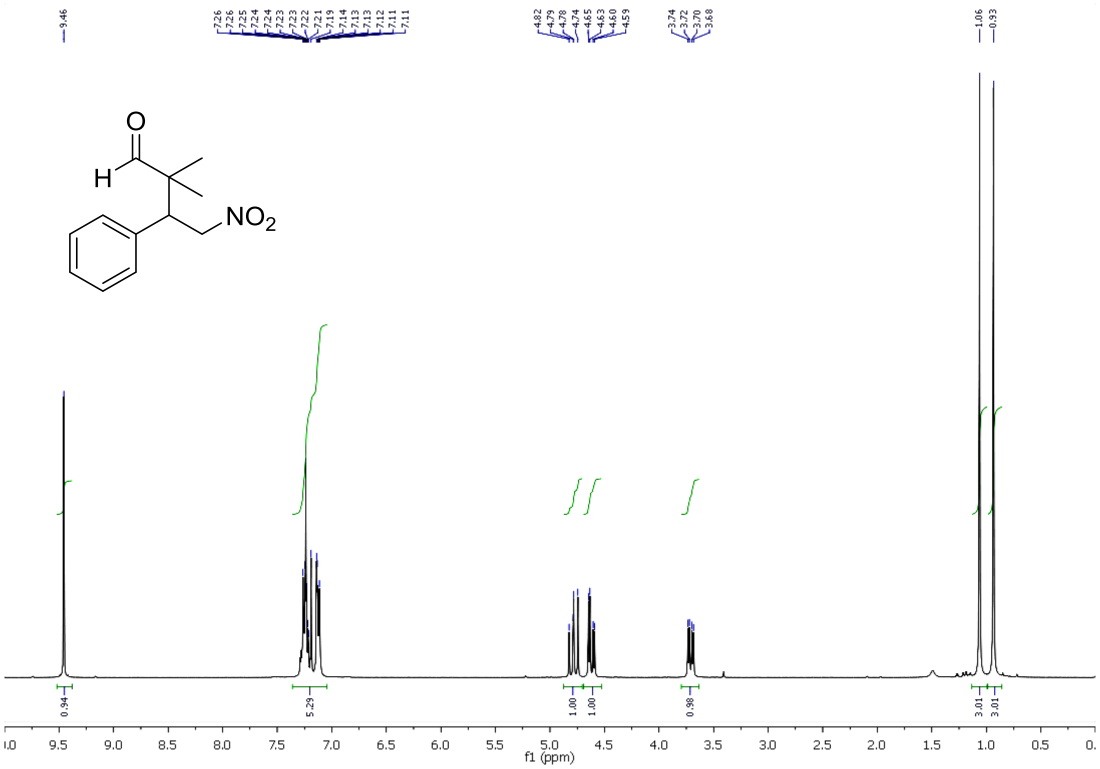

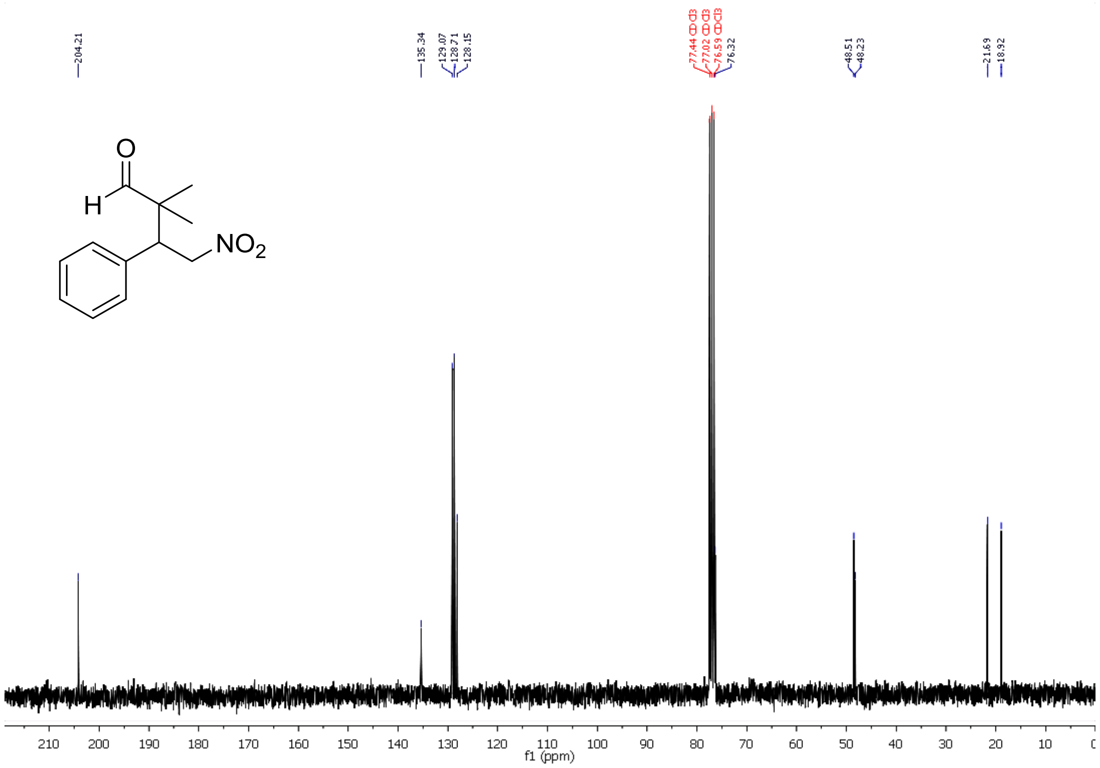

Supplementary Figure 28. ^{1}H NMR and ^{13}C NMR of the Michael-type addition product of isobutyraldehyde to *trans*-β-nitrostyrene.

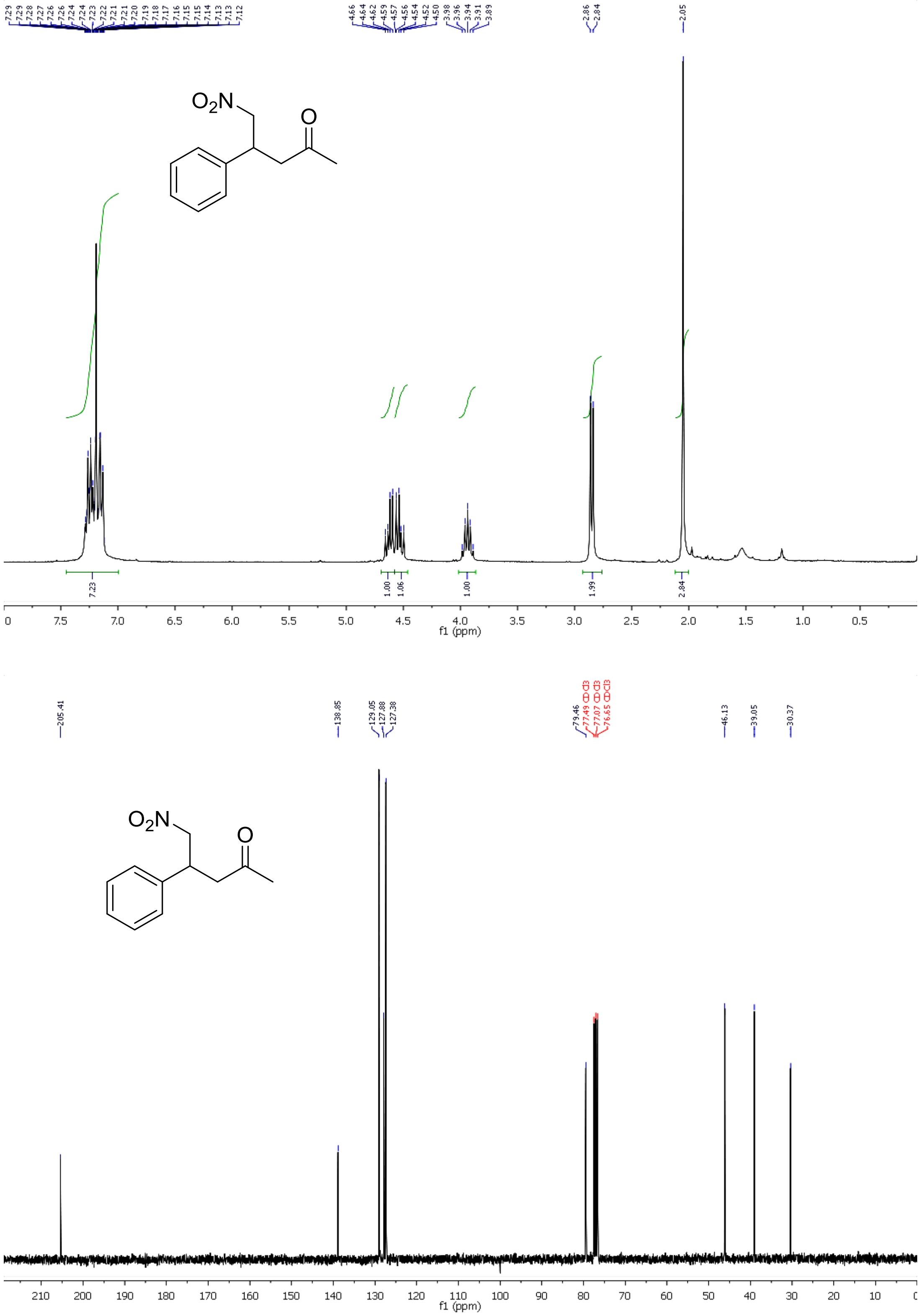

Supplementary Figure 29. ^{1}H NMR and ^{13}C NMR of the Michael-type addition product of nitromethane to 4-phenyl-3-buten-2-one.

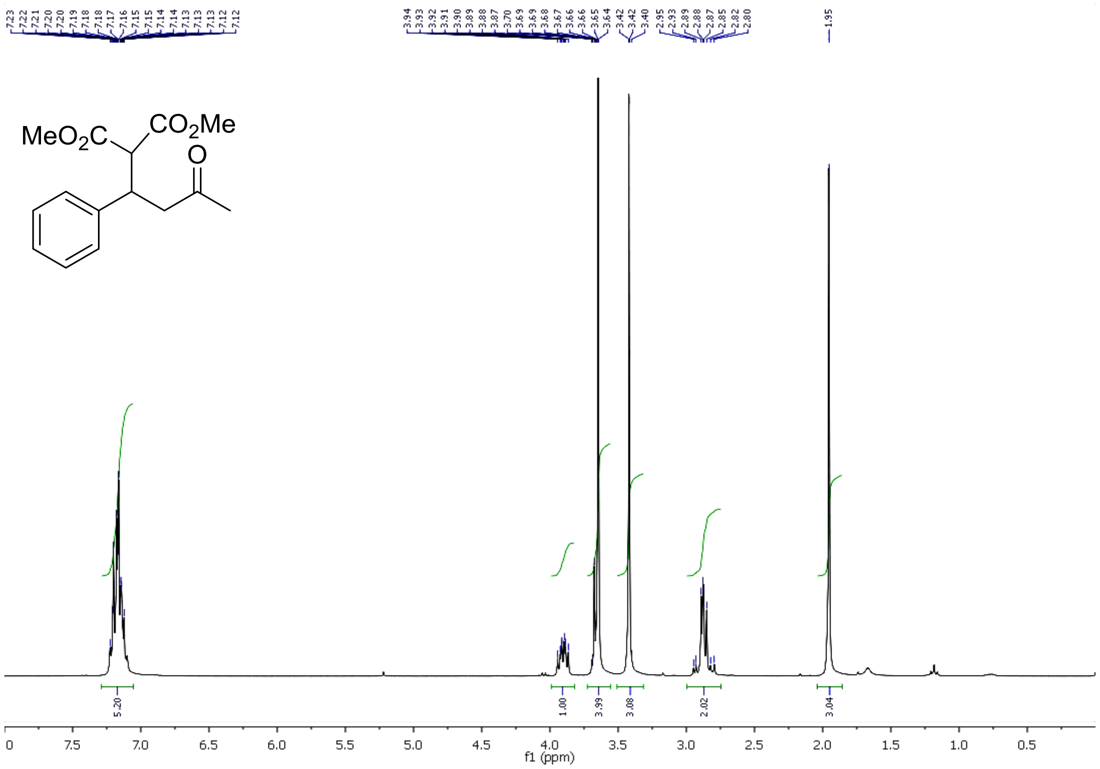

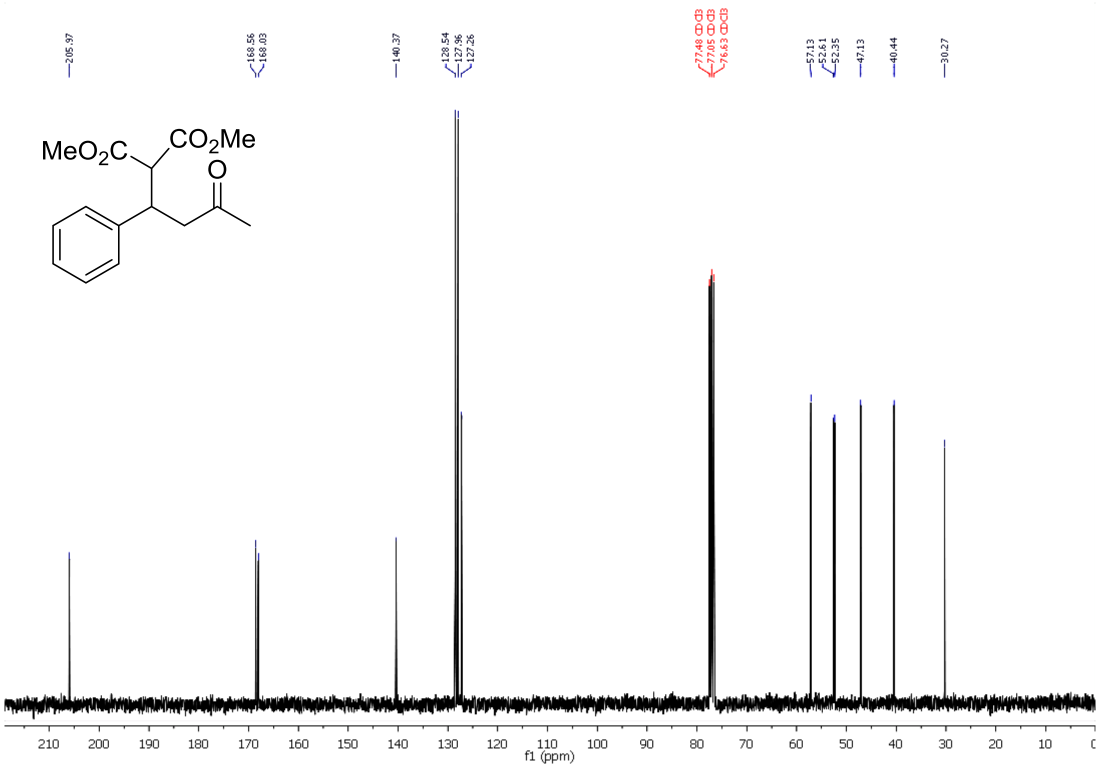

Supplementary Figure 30. ^{1}H NMR and ^{13}C NMR of the Michael-type addition product of dimethylmalonate to 4-phenyl-3-buten-2-one.

Area % Report

Data File: C:\EZChrom Elite\Enterprise\Projects\Default\Data\PilarG\2015\04\PGGXII-16-04 02-04-2015 18-40-03.dat
Method: C:\EZChrom Elite\Enterprise\Projects\Default\Method\MariaBG 100 CH3CN 0.1 OVERNIGHT .met
Acquired: 02/04/2015 18:41:27

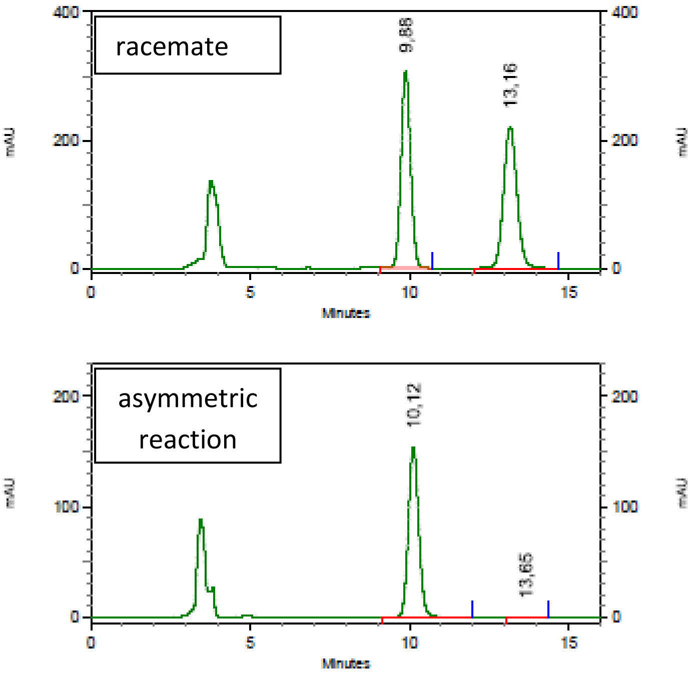

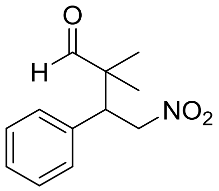

VWD: Signal A, 220 nm Results

Retention Time	Area	Area %
10,123	58879681	99,18
13,650	486543	0,82
Totals	59366224	100,00

Supplementary Figure 31. HPLC traces of the Michael-type addition product of isobutyraldehyde to *trans*-β-nitrostyrene. Conditions: Kromasil 5-Cellucoat, n-hexane/isopropanol = 70:30, 1mL/min, 220 nm.

Area % Report

Data File: C:\EZChrom Elite\Enterprise\Projects\Default\Data\PilarG\2015\03\PGGXI-74-14 04-03-2015 16-14-14.dat
Method: C:\EZChrom Elite\Enterprise\Projects\Default\Method\MariaBG 100 CH3CN 0.1 OVERNIGHT .met
Acquired: 04/03/2015 16:15:34

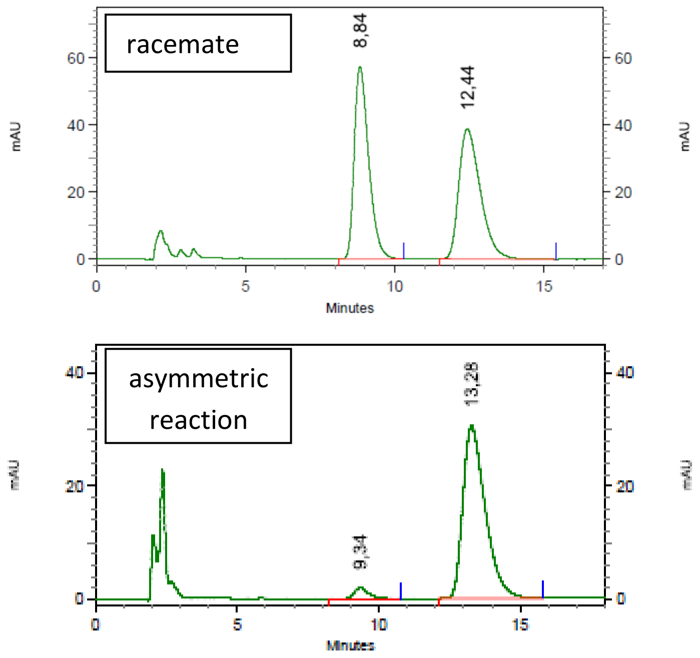

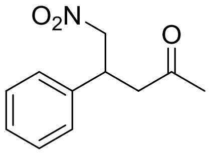

VWD: Signal A, 220 nm Results

Retention Time	Area	Area %
9,340	1187090	4,04
13,283	28213543	95,96
Totals	29400633	100,00

Supplementary Figure 32. HPLC traces of the Michael-type addition product of nitromethane to 4-phenyl-3-buten-2-one. Conditions: Chiralcel OJ, n-hexane/isopropanol = 40:60, 1mL/min, 220 nm.

Area % Report

Data File: C:\EZChrom Elite\Enterprise\Projects\Default\Data\PilarG\2015\03\PGGXI-74-12 04-03-2015 13-04-37.dat
Method: C:\EZChrom Elite\Enterprise\Projects\Default\Method\MariaBG 100 CH3CN 0.1 OVERNIGHT .met
Acquired: 04/03/2015 13:05:58

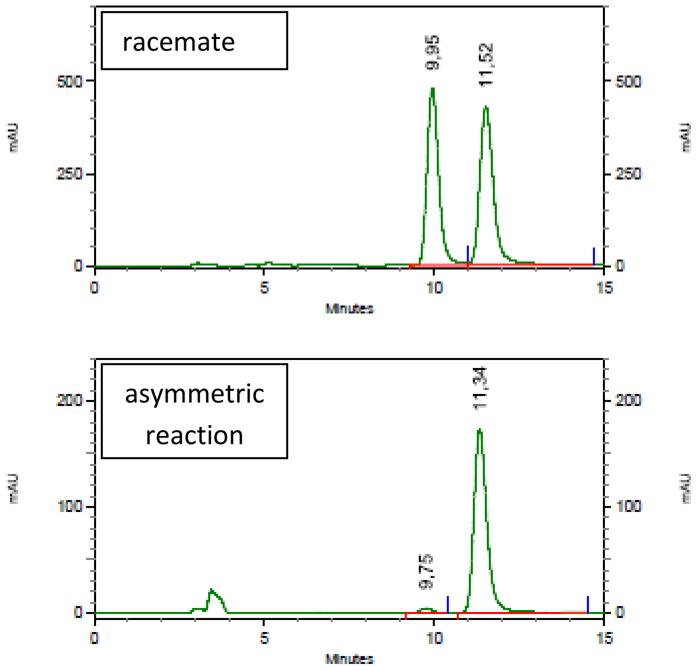

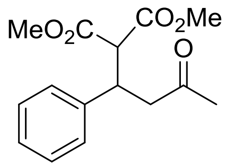

VWD: Signal A, 220 nm Results

Retention Time	Area	Area %
9,750	1790949	2,25
11,340	77882937	97,75
Totals	79673886	100,00

Supplementary Figure 33. HPLC traces of the Michael-type addition product of dimethylmalonate to 4-phenyl-3-buten-2-one. Conditions: Kromasil 5-Amycoat, n-hexane/isopropanol = 90:10, 1mL/min, 220 nm.

Supplementary Tables

Supplementary Table 1. Chemical analysis of Al-ITQ-HB sample. From the elemental CHNS analysis shown, it was possible to estimate the amount of organic content present in the mesoscopic hybrid material, Al-ITQ-HB, incorporated in each individual metalorganic nanolayers from the organic spacers (HB) used during the synthesis process. The results indicated that the organic counterpart contribution was around 40% wt, corresponding to alkyl arylic fragments of the HB compounds. Furthermore, the practical absence of nitrogen content in the solid showed that all dimethylformamide (DMF), used as solvent during the solvothermal synthesis, was completely removed during the successive washing steps.

				**Org.Cont.**[a]	
**Sample**	**C**[a]	**H**[a]	**N**[a]	**CHN**[b]	**TGA**[c]
Al-ITQ-HB	34.3	5.6	0.3	40.2	54.1

[a]Percentage in weight total (%wt); [b]Organic content from CHNS elemental analysis, [c]Organic content from thermogravimetrical analysis (Supplementary Fig. 4) without taking into account hydration water.

Supplementary Table 2. Screening of Al-ITQ-HB loading in the Knoevenagel condensation of 2-naphthaldehyde 1a with Meldrum's Acid 2. Reaction conditions: the mesoscopic hybrid material, Al-ITQ-HB (as in the indicated amount) or the corresponding catalyst (as in the indicated amount was placed in a 1 mL glass vessel. Aldehyde **1a** (0.1 mmol, 15.6 mg) and compound **2** (0.1 mmol, 14.4 mg) were then added. Toluene (50 µL) and water (150 µL) were subsequently added and the reaction mixture was left to stir vigorously at room temperature for 3 h. The product was then extracted with EtOAc (3 x 1 mL), and the solvent evaporated *in vacuo*. Products were analyzed by ^{1}H NMR and yield determined by using triphenylmethane as internal standard.

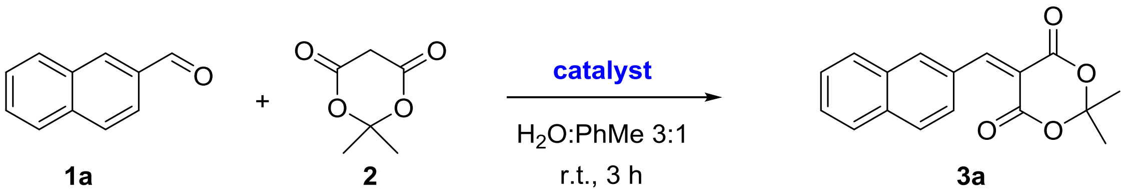

Entry	catalyst (mol%)	Yield [%]
1	---	1
2	Al-ITQ-HB (5)	21
3	Al-ITQ-HB (10)	35
4	Al-ITQ-HB (20)	48
5	Al-ITQ-HB (30)	62
6	Mil-53 (Al) (20)	5

Supplementary Table 3. Solvent screening in the Knoevenagel condensation of 2-naphthaldehyde 1a with Meldrum's Acid 2. Reaction conditions as in Supplementary Table 2. Experiments were carried out with 10 mol% of hybrid mesoscopic catalyst Al-ITQ-HB for 5 hours. Chloroform was the solvent of choice for the Knoevenagel condensation.

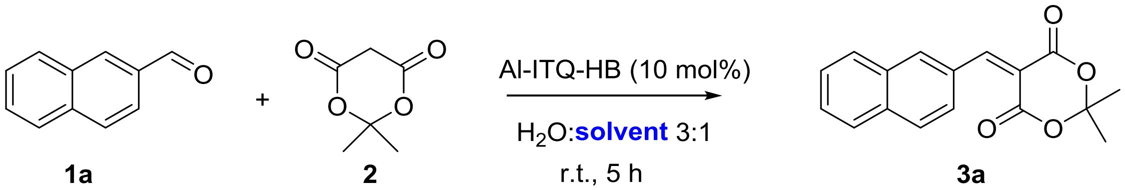

Entry	solvent	Yield [%]
1	PhMe	47
2	ACN	51
3	CH_2Cl_2	62
**4**	**$CHCl_3$**	**68**
5	MeOH	50
6	DMF	68
7	DMSO	51
8	1,4-dioxane	63
9	THF	53
10	Et_2O	44
11	AcOEt	38
12	hexane	49
13	$MeNO_2$	40
14	H_2O	36

Supplementary Table 4. Knoevenagel condensation of 2-naphthaldehyde 1a with Meldrum's Acid 2. The results showed that 20 or 30 mol% of host catalyst Al-ITQ-HB produced good yield of product **3a** after 10 hours. This reaction conditions were used to evaluate the substrate scope.

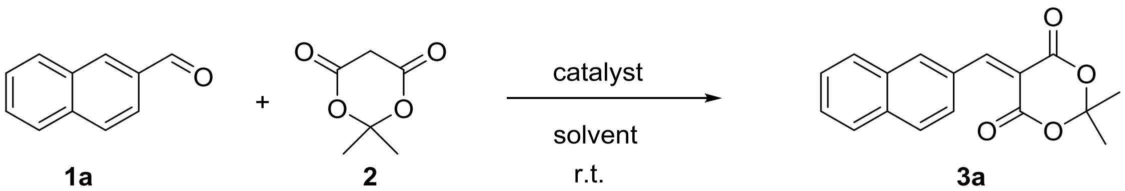

Entry	Catalyst (mol%)	time (h)	Solvent	Yield (%)
1	---	10	H_2O:$CHCl_3$ 1:1 (0.5 M)	4
2	Al-ITQ-HB (10)	10	H_2O:$CHCl_3$ 1:1 (0.5 M)	65
3	Al-ITQ-HB (20)	10	H_2O:$CHCl_3$ 1:1 (0.5 M)	87
4	Al-ITQ-HB (30)	10	H_2O:$CHCl_3$ 1:1 (0.5 M)	91
5	Mil-53 (Al) (30)	10	H_2O:$CHCl_3$ 1:1 (0.5 M)	17
6	---	10	$CHCl_3$ (0.5 M)	1
7	---	10	$CHCl_3$ (1.0 M)	4
8	---	10	$CHCl_3$ (2.0 M)	10
9	Al-ITQ-HB (10)	10	$CHCl_3$ (2.0 M)	27
10	Al-ITQ-HB (20)	10	$CHCl_3$ (2.0 M)	70
11	Al-ITQ-HB (30)	10	$CHCl_3$ (2.0 M)	83
12	Mil-53 (Al) (30)	10	$CHCl_3$ (2.0 M)	18
13	$Al(OH)(C_2H_3O_2)_2$ (30)	10	H_2O:$CHCl_3$ 1:1 (0.5 M)	15
14	4-heptylbenzoic acid (30)	10	H_2O:$CHCl_3$ 1:1 (0.5 M)	21
15	$Al(OH)(C_2H_3O_2)_2$ (30)	10	$CHCl_3$ (2.0 M)	15
16	4-heptylbenzoic acid (30)	10	$CHCl_3$ (2.0 M)	38
17	sodium 4-heptylbenzoate (30)	10	H_2O:$CHCl_3$ 1:1 (0.5 M)	19

Supplementary Table 5. Enantioselective organocatalytic Michael addition of isobutyraldehyde to nitrostyrene. Reaction conditions: the mesoscopic hybrid material, Al-ITQ-HB (8 mg, or the indicated amount) or the corresponding additive (as indicated below) was placed in a 1 mL glass vessel. Organocatalyst **4** (4.7 mg, 0.01 mmol) and *trans*-β-nitrostyrene (15.0 mg, 0.1 mmol) were then added. Toluene (50 µL) and water (150 µL) or the corresponding solvent (as indicated below) were subsequently added followed by isobutyraldehyde (27 µL, 0.3 mmol) and the reaction mixture was left to stir vigorously at room temperature for 24 h. The product was extracted with EtOAc (3 x 1 mL), and was analyzed by GC using dodecane as internal standard. Enantiomeric ratios were determined after column chromatography using HPLC on a chiral stationary phase.

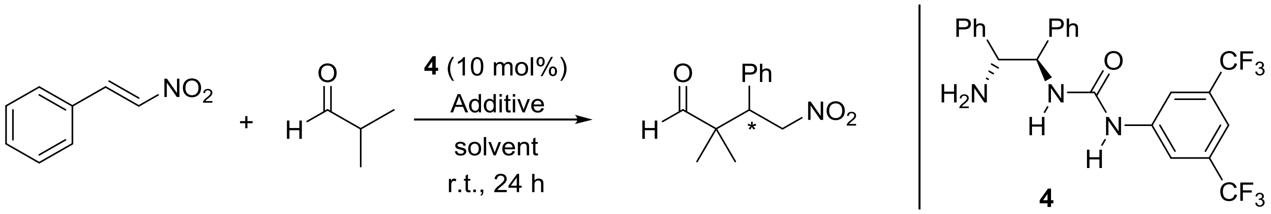

Entry	Additive	Solvent	Yield [%]	ee [%]
1	---	PhMe:H_2O 1:3	14	99
2	Al-ITQ-HB (30 mol%)	PhMe:H_2O 1:3	96	98
3	$C_7H_{15}C_6H_5$ (30 mol%)	PhMe:H_2O 1:3	16	99
4	$C_7H_{15}C_6H_4CO_2H$ (30 mol%)	PhMe:H_2O 1:3	15	99
5	$C_7H_{15}C_6H_4CO_2H$ (20 mol%)	PhMe:H_2O 1:3	18	99
6	$C_7H_{15}C_6H_4CO_2H$ (10 mol%)	PhMe:H_2O 1:3	28	99
7	$C_7H_{15}C_6H_4CO_2H$ (5 mol%)	PhMe:H_2O 1:3	50	99
8	$C_7H_{15}C_6H_4CO_2H$ (2.5 mol%)	PhMe:H_2O 1:3	94	99
9	Mil-53(Al) (30 mol%)	PhMe:H_2O 1:3	15	98
10	---	PhMe	27	99
11	Al-ITQ-HB (30 mol%)	PhMe	50	96
12	Al-ITQ-DB (30 mol%)	PhMe	50	98

13	---	CH_2Cl_2	43	97
14	Al-ITQ-HB (30 mol%)	CH_2Cl_2	63	98
15	Al-ITQ-DB (30 mol%)	PhMe:H_2O 1:3	95	98
16	$C_7H_{15}C_6H_4CO_2Na$ (30 mol%)	PhMe:H_2O 1:3	88	98
17	$C_{12}H_{25}C_6H_4CO_2Na$ (30 mol%)	PhMe:H_2O 1:3	91	98
18	Al-ITQ-HB (7 mol%)	PhMe:H_2O 1:3	77	99
19	Al-ITQ-HB (15 mol%)	PhMe:H_2O 1:3	82	98
20	Al-ITQ-HB (23 mol%)	PhMe:H_2O 1:3	88	98
21	---	brine:H_2O 1:3	41	99
22	Al-ITQ-HB (30 mol%)	brine:H_2O 1:3	95	98

Supplementary Methods

1. General Experimental Information.

Unless otherwise stated, all reagents were purchased from commercial suppliers and used without further purification. Solvents employed in the reactions were purified using a solvent purification system (SPS) MBraun 800. Organic solutions were concentrated under reduced pressure on a Büchi rotary evaporator. Reactions were monitored by thin layer chromatography on silica gel pre-coated aluminium plates using fluorescence quenching with UV light at 254 nm or $KMnO_4$. Flash column chromatography was performed using E. Merck silica gel (60, particle size 0.040-0.063 mm). Chemical yields refer to pure isolated substances unless stated otherwise. All the products obtained were characterised by IR, GC-MS, ^{1}H- and ^{13}C-NMR, and DEPT. Characterisation given in the literature was used for comparison.[2-4] Gas chromatographic analyses were performed in an instrument equipped with a 25 m capillary column of 5% phenylmethylsilicone using dodecane as an external standard otherwise indicated. GC/MS analyses were performed on a spectrometer equipped with the same column as the GC and operated under the same conditions.^{1}H and ^{13}C NMR were recorded on a Bruker 300 spectrometer and the chemical shifts are reported in ppm relative to residual proton solvents signals. Data for ^{1}H NMR spectra are reported as follows: chemical shift (δ, ppm), multiplicity (s = singlet, d = doublet, t = triplet, q = quartet, m = multiplet, dd = double doublets), coupling constant and integration. Data for ^{13}C NMR spectra are reported in chemical shift (δ, ppm). High performance liquid chromatography (HPLC) was performed on Agilent Technologies 1220 Infinity Series instrument using a Daicel Chiralcel OJ (4.6 x 250 mm), Kromasil 5-AmyCoat (4.6 x 250 mm) and Kromasil 5-CelluCoat (4.6 x 250 mm). C, N, and H contents were determined with a Carlo Erba 1106 elemental analyzer. Thermogravimetric and differential thermal analysis (TGA-DTA) were conducted in an air stream with a Metler Toledo TGA/SDTA 851E analyzer. Solid state MAS-NMR spectra were recorded at room temperature under magic angle spinning (MAS) in a Brucker AV-400 spectrometer. The ^{13}C cross-polarization (CP) spectrum was acquired by using a 7 mm Bruker BL-7 probe and at a sample spinning rate of 5kHz. ^{13}C was referred to adamantane. Solution saturation-transfer difference (STD) and NOESY NMR spectroscopy experiments were performed at 25 ºC in a Bruker Avance 800 MHz spectrometer equipped with inverse triple-resonance TCI cryo-probe and pulse gradients. NMR samples were prepared in 0.5 mL of $CDCl_3$. STD-NMR experiments were performed using pulse sequences reported in the literature.[5,6] Spectra were recorded using a train of 50 ms Gaussian shaped pulses with flipping angles of 650º (height 87.7 Hz), 2.0 s of total irradiation time. The on-resonance frequency was set to 0.9 ppm or 7.2 ppm (indicated in each case), whereas off-resonance frequency

was -150 ppm. Blank experiments were performed to assure the absence of direct saturation to/of the ligand protons. NOESY experiments were performed with 50 or 150 ms mixing time (indicated in each case). In these conditions, 800 MHz and 50 ms mixing time, NOESY spectra of free and monomeric substrates do not show observable NOE signals.

FTIR spectra were recorded with a Nicolet 710 spectrometer (4 cm^{-1} resolution) using conventional greaseless cell. IR spectra of the organic precursors were recorded on KBr disks at room temperature or by impregnating the windows with a dichloromethane solution of the compound and leaving to evaporate before analysis. Ar adsorption isotherm was performed at 87.3 K in an ASAP 2010 apparatus from Micromeritics, after pretreating the samples under vacuum at 333 K overnight. Hörvath-Kawazoe model was used to estimate pore size distribution.[7,8]

2. Characterization of Compounds

2,2-Dimethyl-5-(naphthalen-2-ylmethylene)-1,3-dioxane-4,6-dione (3a)[2]

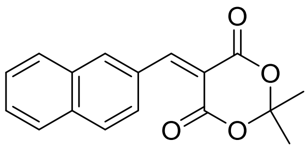

^{1}H-NMR (300 MHz, $CDCl_3$): δ 8.61 (1H, s), 8.57 (1H, s), 8.15 (1H, d, J = 8.7 Hz), 7.97 (1H, d, J = 7.9 Hz), 7.90 (1H, d, J = 8.7 Hz), 7.89 (1H, d, J = 8.1 Hz), 7.65 (1H, dd, J = 6.8 and 8.0 Hz), 7.58 (1H, dd, J = 8.0 and 6.9 Hz), 1.86 (6H, s).

2,2-Dimethyl-5-(naphthalen-1-ylmethylene)-1,3-dioxane-4,6-dione (3b)[2]

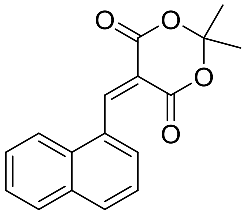

^{1}H-NMR (300 MHz, $CDCl_3$): δ 9.14 (1H, s), 7.94 (2H, d, J = 7.7 Hz), 7.90 (1H, d, J = 8.9 Hz), 7.85 (1H, d, J = 7.7 Hz), 7.54 (1H, t. J = 8.8 Hz), 7.50 (1H, t, J = 7.7 Hz), 7.46 (1H, t, J = 8.8 Hz), 1.79 (6H, s).

5-benzylidene-2,2-dimethyl-1,3-dioxane-4,6-dione (3c)[2]

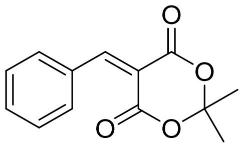

^{1}H-NMR (300 MHz, $CDCl_3$): δ 8.36 (1H, s), 7.98 (2H, d, J = 7.7 Hz), 7.50 (1H, t, J = 7.6 Hz), 7.42 (2H, t, J = 7.7 Hz), 1.74 (6H, s).

5-(2-methoxybenzylidene)-2,2-dimethyl-1,3-dioxane-4,6-dione (3d)[3]

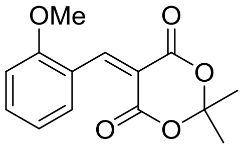

^{1}H-NMR (300 MHz, $CDCl_3$): δ 8.66 (1H, s), 7.88 (1H, d, J = 8.0 Hz), 7.43 (1H, t, J = 8.7 Hz), 6.94 (1H, t, J = 8.0 Hz), 6.87 (1H, d, J = 8.4 Hz), 3.82 (3H, s), 1.74 (6H, s).

5-(4-chlorobenzylidene)-2,2-dimethyl-1,3-dioxane-4,6-dione (3e)[4]

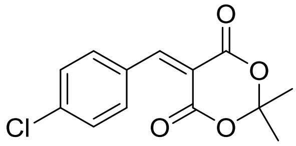

^{1}H-NMR (300 MHz, $CDCl_3$): δ 8.30 (1H, s), 7.95 (2H, d, J = 8.7 Hz), 7.39 (2H, d, J = 8.5 Hz), 1.74 (6H, s).

2-Amino-5-oxo-7,7-dimethyl-spiro[(4*H*)-5,6,7,8-tetrahydrochromene-4,3′-(3′*H*)-indol]-(1′*H*)-2′-one-3-carbonitrile[9]

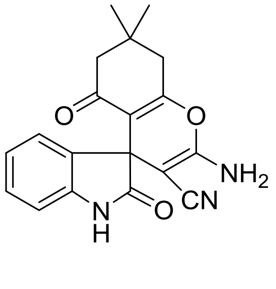

^{1}H-NMR (300 MHz, d6-DMSO): δ 10.39 (1H, s), 7.22 (2H, br s), 7.14 (1H, t, J = 7.6 Hz), 6.98 (1H, d, J = 7.8 Hz), 6.89 (1H, t, J = 7.4 Hz), 6.79 (1H, d, J = 7.7 Hz), 2.56-2.49 (2H, m), 2.18 (1H, d, J = 16.1 Hz), 2.09 (1H, d, J = 16.0 Hz), 1.03 (3H, s), 1.00 (3H, s). ^{13}C NMR (75 MHz, d6-DMSO): δ 194.8, 177.9, 164.1, 158.7, 142.0, 134.4, 128.1, 123.0, 121.6, 117.3, 110.7, 109.2, 57.5, 50.0, 46.8, 39.9, 31.9, 27.6, 27.0.

2,2-dimethyl-4-nitro-3-phenylbutanal[10]

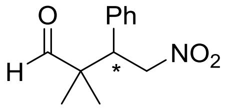

^{1}H-NMR (300 MHz, $CDCl_3$): δ 9.46 (s, 1H), 7.51 – 6.94 (m, 5H), 4.78 (dd, J = 13.1, 11.2 Hz, 1H), 4.62 (dd, J = 13.0, 4.3 Hz, 1H), 3.71 (dd, J = 11.2, 4.3 Hz, 1H), 1.06 (s, 3H), 0.93 (s, 3H). ^{13}C NMR (75 MHz, $CDCl_3$): δ 204.2, 135.3, 129.1, 128.7, 128.2, 76.3, 48.5, 48.2, 21.7, 18.9.

dimethyl 2-(3-oxo-1-phenylbutyl)malonate[11]

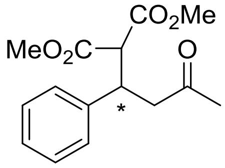

^{1}H-NMR (300 MHz, $CDCl_3$): δ 7.35 – 6.87 (m, 5H), 3.91 (ddd, J = 9.8, 8.2, 5.5 Hz, 1H), 3.66 (d, J = 8.3 Hz, 1H), 3.65 (s, 3H), 3.42 (s, 3H), 3.04 – 2.71 (m, 2H), 1.95 (s, 3H). ^{13}C NMR (75 MHz, $CDCl_3$): δ 206.0, 168.6, 168.0, 140.4, 128.5, 128.0, 127.3, 57.1, 52.6, 52.4, 47.1, 40.4, 30.3.

5-nitro-4-phenylpentan-2-one[12]

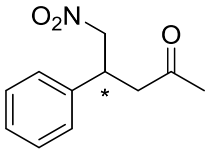

^{1}H-NMR (300 MHz, $CDCl_3$): δ 7.43 – 7.02 (m, 5H), 4.63 (dd, J = 12.3, 6.9 Hz, 1H), 4.53 (dd, J = 12.4, 7.6 Hz, 1H), 3.94 (p, J = 7.1 Hz, 1H), 2.85 (d, J = 7.0 Hz, 2H), 2.05 (s, 3H). ^{13}C NMR (75 MHz, $CDCl_3$): δ 205.4, 138.8, 129.1, 127.9, 127.4, 76.5, 46.1, 39.1, 30.4.

Supplementary References

1 Loiseau, T. *et al.* MIL-96, a Porous Aluminum Trimesate 3D Structure Constructed from a Hexagonal Network of 18-Membered Rings and μ3-Oxo-Centered Trinuclear Units. *J. Am. Chem. Soc.* **128**, 10223-10230, (2006).

2 Murase, T., Nishijima, Y. & Fujita, M. Cage-Catalyzed Knoevenagel Condensation under Neutral Conditions in Water. *J. Am. Chem. Soc.* **134**, 162-164, (2012).

3 Fillion, E., Carret, S., Mercier, L. G. & Trépanier, V. É. Sequential Rh(I)/Pd-Catalyzed 1,4-Addition/Intramolecular Allylation: Stereocontrolled Construction of γ-Butyrolactones and Cyclopropanes. *Org. Lett.* **10**, 437-440, (2008).

4 Fillion, E., Dumas, A. M. & Hogg, S. A. Modular Synthesis of Tetrahydrofluorenones from 5-Alkylidene Meldrum's Acids. *J. Org. Chem.* **71**, 9899-9902, (2006).

5 Mayer, M. & Meyer, B. Characterization of Ligand Binding by Saturation Transfer Difference NMR Spectroscopy. *Angew. Chem. Int. Ed.* **38**, 1784-1788, (1999).

6 Xia, Y., Zhu, Q., Jun, K.-Y., Wang, J. & Gao, X. Clean STD-NMR spectrum for improved detection of ligand-protein interactions at low concentration of protein. *Magnetic Resonance in Chemistry* **48**, 918-924, (2010).

7 Saito, A. & Foley, H. C. Argon porosimetry of selected molecular sieves: experiments and examination of the adapted Horvath-Kawazoe model. *Microporous Materials* **3**, 531-542, (1995).

8 Horvath, G. & Kawazoe, K. Method for the calculation of effective pore size distribution in molecular sieve carbon. *J. Chem. Eng. Jpn.* **16**, 470-475, (1983).

9 Wang, L.-M. *et al.* Sodium stearate-catalyzed multicomponent reactions for efficient synthesis of spirooxindoles in aqueous micellar media. *Tetrahedron* **66**, 339-343, (2010).

10 Avila, A., Chinchilla, R., Fiser, B., Gómez-Bengoa, E. & Nájera, C. Enantioselective Michael addition of isobutyraldehyde to nitroalkenes organocatalyzed by chiral primary amine-guanidines. *Tetrahedron-Asymmetr.* **25**, 462-467, (2014).

11 Dudziński, K., Pakulska, A. M. & Kwiatkowski, P. An Efficient Organocatalytic Method for Highly Enantioselective Michael Addition of Malonates to Enones Catalyzed by Readily Accessible Primary Amine-Thiourea. *Org. Lett.* **14**, 4222-4225, (2012).

12 Ballini, R. *et al.* Polystyryl-BEMP as an Efficient Recyclable Catalyst for the Nucleophilic Addition of Nitroalkanes to α,β-Unsaturated Carbonyl Compounds under Solvent-Free Conditions. *Adv. Synth. Catal.* **350**, 1218-1224, (2008).
